# A LoRa-Based Wireless Sensor Network for Urban Water Quality Monitoring with Real-Time Machine Learning and Energy Analysis

**DOI:** 10.3390/s26196159

**Published:** 2026-09-29

**Authors:** Iman Valizadeh, Lorena Parra, Jaime Lloret

**Affiliations:** 1 Department of Communications, Universitat Politècnica de València, 46022 Valencia, Spain; 2 Departamento de Producción Agraria, Escuela Técnica Superior de Ingeniería Agronómica, Alimentaria y de Biosistemas, Universidad Politécnica de Madrid, 28040 Madrid, Spain

**Keywords:** LoRa-based monitoring, urban water quality, machine learning, Random Forest, UV reconstruction, wireless sensor networks, energy-efficient monitoring

## Abstract

This paper presents a LoRa-based system for real-time urban water quality monitoring. The proposed platform integrates several sensors, including ultraviolet (UV), infrared (IR), red-green-blue (RGB), pH, turbidity, and temperature sensors, to monitor different water quality parameters. A total of 1443 sensor records were collected during the experiments. Among these records, 75 UV measurements, corresponding to approximately 5.2% of the dataset, were missing or invalid while the Red, Green, and Blue (RGB) and IR measurements were available. Several regression and machine learning models were evaluated to reconstruct these UV values. Multiple Linear Regression showed limited performance in the initial random train–test evaluation (R^2^ = 0.12), while second-order Polynomial Regression achieved a considerably higher R^2^ of 0.963. Support Vector Regression (SVR), k-Nearest Neighbors (KNN), Multi-Layer Perceptron (MLP), and Random Forest (RF) were also evaluated. Random Forest obtained the highest R^2^ in the random train–test evaluation (R^2^ = 0.991). However, the results were less consistent when the models were evaluated using Leave-One-Session-Out (LOSO) validation. In this evaluation, KNN obtained the lowest mean absolute error (MAE) and root mean squared error (RMSE), while the mean R^2^ values were negative for all evaluated models, showing limited generalization between experimental sessions. The possible energy benefit of UV reconstruction was also estimated. Based on an active power of 2.995 W and an acquisition time of 10 s, avoiding 75 additional acquisition cycles would correspond to approximately 0.624 Wh of acquisition energy, or 4.94% under the assumptions used in the energy analysis. These results show that machine learning can be useful for reconstructing individual missing or invalid UV measurements when the corresponding optical measurements are available. At the same time, the differences observed between experimental sessions show that further experiments with more independent sessions and longer field deployments are needed.

## 1. Introduction

In recent years, monitoring water quality and addressing environmental challenges caused by industrial and agricultural pollutants have become major concerns for scientists and researchers worldwide [1]. This issue is particularly important in large cities, where groundwater and surface water resources are strongly affected by human activities. The increase in urbanization and industrial development has made water pollution control even more challenging [2,3]. Untreated urban and industrial discharges remain among the main sources of surface water pollution [4,5]. Therefore, the need for innovative solutions for accurate and continuous monitoring of these resources has become increasingly important to protect both public health and the environment [6,7]. According to a report published in the Journal of Global Health, more than one million people die every year due to unsafe water and poor sanitation, while hundreds of millions suffer from water-related diseases [8]. As a result, Wireless Sensor Networks (WSNs) have been considered a promising alternative to complement traditional monitoring processes. These networks are relatively cost-effective and allow measurements to be taken remotely, in real time, and with minimal human intervention [9,10]. Among the available communication technologies, LoRa has been introduced as an efficient and low-cost solution for large-scale data transmission in urban environments. With its long communication range and low energy consumption, it enables continuous and real-time monitoring [11].

However, practical sensing systems may contain missing or invalid sensor measurements due to communication instability or temporary sensing faults [12]. In the collected dataset, some partially received records contained missing or invalid UV measurements while the corresponding RGB and IR measurements remained available. In this study, UV intensity was selected as a sensitive optical indicator because of its correlation with RGB and IR measurements. This characteristic makes it suitable for predictive reconstruction when individual UV measurements are missing or invalid while the corresponding RGB and IR measurements remain available. To address this issue, machine learning models were applied to estimate missing UV values and improve the reliability of the collected environmental data. The deployment of this system supports continuous water quality monitoring and provides valuable insight into variations in monitored water conditions. By combining Internet of Things (IoT) technologies, LoRa communication, and machine learning, this study proposes a practical framework for continuous and intelligent water quality monitoring. This study aims to design an innovative five-node LoRa-based sensing framework for accurate water quality monitoring in an urban environment. The developed platform integrates multiple sensors, including pH, temperature, turbidity, RGB, IR, and UV, enabling precise measurement of key water quality parameters [13]. In the present experimental phase, one sensing node was physically implemented and validated under real urban monitoring conditions, while the remaining nodes represent the scalable deployment architecture of the proposed network.

Recent advancements have further emphasized the integration of edge computing and dynamic LoRa network topologies to optimize energy consumption and enable reliable, real-time water quality monitoring [14,15]. Furthermore, addressing the persistent challenge of missing sensor data through advanced machine learning imputation techniques has become a critical focus for ensuring the resilience, continuity, and accuracy of IoT-based aquatic monitoring systems [16,17]. The present study builds upon these emerging trends by proposing a unified framework that combines edge-level predictive reconstruction with energy analysis.

It should be noted that the proposed system is intended as a multi-sensor monitoring and screening platform rather than a replacement for comprehensive laboratory-based chemical analysis. The selected sensing parameters provide complementary information on physical and optical variations in water and can support continuous detection of changes in water conditions. Near-infrared (NIR) sensors, such as the AS7263, could also be considered as an additional sensing option for the proposed system. Although this sensor was not used or experimentally evaluated in the current setup, it could provide additional spectral information to complement the existing measurements. Therefore, the integration and experimental evaluation of NIR sensing can be considered in future work. However, the identification and quantification of specific chemical contaminants may require dedicated analytical or spectroscopic techniques. Therefore, the proposed architecture is designed primarily for continuous monitoring and early detection of abnormal changes, while laboratory analysis may be required for definitive chemical characterization.

A total of 1443 sensor records were collected from the implemented sensing node under urban monitoring conditions in Gandia. Among these samples, approximately 5.2% of the UV measurements were missing, which motivated the implementation of the proposed reconstruction framework. Several regression and machine learning methods were evaluated to estimate these missing values and reduce the potential need for additional data reacquisition.

The following sections present the sensing architecture, data preprocessing steps, machine learning workflow, and experimental analysis in detail. The results demonstrate how predictive reconstruction can support data reliability and contribute to the overall efficiency of the monitoring system. The remainder of this paper is organized as follows. Section 2 reviews the related literature. Section 3 describes the sensing platform, deployment conditions, data acquisition process, and the proposed machine learning workflow. Section 4 presents the data analysis, model evaluation, and energy consumption assessment. Section 5 presents the experimental results, including the performance of the machine learning-based data reconstruction approach. Section 6 discusses the findings, limitations, and practical implications of the proposed system. Finally, Section 7 concludes the paper and outlines future research directions.

## 2. Literature Review

This section reviews previous studies on wireless sensor networks and IoT-based systems for water quality monitoring, with particular emphasis on sensing reliability, data analysis methods, and long-range communication technologies. Existing works are examined in terms of sensing accuracy, communication range, data reliability, and practical deployment in urban environments. The limitations identified in these studies highlight the need for an integrated framework that combines reliable long-distance communication with intelligent data reconstruction, which motivates the proposed system.

Wireless Sensor Networks (WSNs) have become an important part of modern urban life, supporting applications such as IoT services, smart city traffic control, and environmental monitoring [18,19]. Water pollution has been recognized as a major global concern due to its environmental, economic, and public health impacts. The quality of water resources in many regions has been severely affected by industrial, agricultural, and urban activities [20]. As a result, accurate and automated monitoring systems have become increasingly necessary. Several studies have therefore explored smart water monitoring using IoT and wireless communication technologies [21]. Recent advances in wireless sensor networks have also emphasized the importance of energy-efficient communication and routing strategies to prolong node lifetime in practical sensing deployments [22]. In addition, scalable localization and network coordination techniques remain important design considerations in advanced wireless sensor network architectures [23]. In ref. [24], a smart water distribution and quality monitoring solution based on machine-to-machine LoRa communication was proposed. Their work focused on pH, turbidity, electrical conductivity, dissolved oxygen, and chemical parameters. However, the study mainly described the system concept and did not provide practical real-world performance validation. Machine learning has also been widely applied in water quality analysis. Ahmed et al. [25] evaluated approximately 660 samples from 13 water sources and applied several machine learning algorithms to estimate water quality parameters. Their best reported accuracy was approximately 85% using a Multi-Layer Perceptron model. Similarly, other studies have demonstrated that machine learning methods can significantly improve water quality prediction accuracy by capturing complex nonlinear relationships among physicochemical parameters [26]. However, their work focused primarily on offline data analysis and did not integrate the models into a real-time sensing platform.

Similarly, the intelligent IoT framework proposed in ref. [27] employed Artificial Neural Networks and K-Means clustering for anomaly detection and water quality classification. Although the study demonstrated the potential of machine learning for decision support, it relied mainly on processed datasets and did not validate the approach in a practical urban sensing environment. A broader AI-based industrial wastewater assessment framework was presented in ref. [28], where several classifiers, including Support Vector Machine, Random Forest, Categorical Boosting (CatBoost), Extreme Gradient Boosting (XGBoost), Decision Tree, and Multi-Layer Perceptron, were evaluated on approximately 1600 samples. CATBoost achieved the best classification performance. Nevertheless, the study did not include hardware deployment or communication-level reliability analysis, leaving uncertainty regarding real-time implementation feasibility. Other studies have focused on data-driven classification without hardware integration. For example, Abuzir and Abuzir [29] analyzed a Kaggle water quality dataset using J48, Naïve Bayes, and MLP models. Their best performance was also obtained with MLP. While their feature engineering and preprocessing methods were comprehensive, the study remained limited to offline dataset analysis without deployment in a real monitoring system. From the communication perspective, Jawad et al. [30] reviewed energy-efficient wireless sensor networks for precision agriculture, highlighting the critical role of low-power, long-range communication protocols in extending node lifetime for environmental monitoring. Similarly, previous LoRa-based systems such as [13] concentrated on hardware implementation using separate sensing and gateway nodes for cloud transmission. While these systems demonstrated the feasibility of practical deployment, they did not investigate missing data handling or predictive reconstruction for improving data continuity.

Data-driven approaches have also been investigated to improve reliability and fault handling in water monitoring systems. Pérez–Pérez et al. [31] proposed a neuro-fuzzy Zonotopic Kalman Filter approach for sensor fault and leak diagnosis in water pipelines using pressure and flow measurements. In contrast to such fault-diagnosis approaches, the present study focuses on reconstructing missing UV measurements using correlated optical sensor data in a LoRa-based monitoring system.

Overall, previous studies have generally focused either on sensor data analysis using machine learning or on hardware implementation for long-range communication. The main gap lies in combining both aspects within a single scalable and practical framework. The present study addresses this gap by designing a five-node LoRa-based sensing architecture for urban deployment and experimentally validating one sensing node under real monitoring conditions. In addition, the proposed approach uses machine learning to reconstruct missing or invalid UV measurements when the corresponding RGB and IR measurements remain available, supporting data continuity within the monitoring system.

## 3. Materials and Methods

The proposed system is an innovative and robust solution for monitoring the health and quality of water in urban water systems. It comprises a scalable architecture of five sensing nodes and one central receiver, forming a wireless sensor network that utilizes LoRa communication technology for long-range and low-power data transmission. In the current experimental phase, one sensing node was physically implemented and validated under real urban monitoring conditions. Each sensing node is equipped with multiple sensors selected to measure key water quality parameters, including UV, IR, turbidity, temperature, pH, and the intensity of red, green, and blue light. The nodes are designed to operate independently and are powered by 10,000 mAh power banks, ensuring flexibility and suitability for scalable field deployment.

The implemented sensing node collects real-time data from urban water sources and transmits this information via the 868 MHz frequency band to the central receiver. The central receiver, equipped with an SD card module, stores the data for subsequent analysis. The use of optical sensors housed within a specialized black plastic frame ensures precise measurements by eliminating ambient light interference. Environmental sensors, such as turbidity, temperature, and pH, complement the optical readings and provide a comprehensive assessment of water quality. This system demonstrates the seamless integration of advanced sensing technologies and efficient communication protocols, offering a scalable and cost-effective approach for monitoring urban water. Its architecture supports real-time data visualization on integrated LCD displays and ensures reliable transmission over significant distances. This design not only improves operational efficiency but also enhances the accessibility of data for real-time decision-making and long-term environmental monitoring.

### 3.1. System Components

The hardware architecture of the system comprises two main components: five sensing nodes (Node1 to Node5) and the receiver, which acts as the gateway. In the current experimental setup, one sensing node was fully implemented and used for data collection and model validation. Each sensing node includes multiple sensors for measuring water quality parameters and is capable of collecting data from the surrounding environment and sending this information to the receiver. The gateway functions as the central hub, receiving data from the sensing nodes and forwarding it for further processing and analysis. This part of the system is specifically designed to ensure effective and continuous communication between the sensing nodes and the control center. In the following subsections, the key components and features of each part are described to clearly illustrate how each component interacts and operates within the overall system.

#### 3.1.1. Transmitters

As mentioned earlier, the overall sensing framework was designed as a scalable five-node architecture for deployment in different parts of the city. In the current experimental phase, one sensing node was physically implemented and validated. Figure 1, shows the structure of this node.

The structure of the implemented sensing node is presented in Figure 1. Its main components are as follows:

1. pH Sensor: E-201-C composite electrode, manufactured by INESA Scientific Instrument (Shanghai, China), used to measure the acidity or alkalinity of water.
2. Temperature Sensor: DS18B20 waterproof digital sensor, Maxim Integrated (San Jose, CA, USA), stainless steel probe with 1-Wire interface, measuring range −55 °C to +125 °C with ±0.5 °C accuracy.
3. RGB led Sensor: TCS3200 color light-to-frequency sensor, AMS-TAOS (Premstaetten, Austria), integrated on a Robotrikh module, operates at 630 nm (Red), 525 nm (Green), and 470 nm (Blue). The receptor element is sensitive to all visible light.
4. Ultraviolet Sensor: UV detection system composed of a 365 nm UV LED emitter and a UV photodiode receiver (equivalent to GUVA-S12SD, Genicom, or ML8511, Lapis Semiconductor), providing analog output proportional to UV intensity.
5. Infrared Sensor: Infrared photodiode receiver with peak sensitivity at 940 nm (equivalent to OSRAM SFH 203 P or Vishay TSOP382), used for detecting infrared light through water samples.
6. Glass pipe: Transparent water flow pipe where samples are passed for testing, ensuring optical alignment between emitters and receivers.
7. LCD: 20 × 4-character display (Winstar Display Co., Ltd., Taichung, Taiwan) with an HD44780-compatible controller and I2C interface, used to display real-time sensor readings.
8. Water outlet pipe: Provides controlled discharge of the tested water after measurements.
9. Turbidity Sensor: SEN0189 optical turbidity sensor, DFRobot (Shanghai, China), measures water clarity in Nephelometric Turbidity Units (NTU).
10. Holder base: Structural support platform to hold all sensors and components in proper alignment.
11. LoRa Wi-Fi Module: Heltec WiFi LoRa 32 (V2), Heltec Automation (Chengdu, China), integrates ESP32 microcontroller, SX1276 LoRa transceiver (868 MHz), and Organic Light-Emitting Diode (OLED) display for long-range wireless communication.
12. Antenna External antenna (868 MHz) provided by Heltec Automation, used to enhance LoRa wireless data transmission.

The node includes an Arduino Mega for processing sensor data and a Heltec WiFi LoRa 868 V2 module for wireless transmission to the central receiver. Although the Heltec WiFi LoRa 868 V2 integrates both Wi-Fi and LoRa capabilities, LoRa was used as the primary wireless communication technology between the sensing node and the gateway. The Wi-Fi interface was not required for regular node-to-gateway data transmission and can remain disabled when not needed to avoid unnecessary power consumption. Multiple parameters are measured, including pH, temperature, turbidity, UV, IR, and RGB. The optical sensors are housed in a black plastic enclosure to prevent interference from ambient light. This is particularly important because RGB and IR measurements are later used to reconstruct missing or invalid UV measurements when the corresponding RGB and IR measurements remain available.

Each node operates with a 10,000 mAh power bank, enabling extended field operation. A local display is also integrated for real-time visualization of sensor readings, allowing immediate monitoring and response. As shown in Figure 1, all sensors are electrically connected to the Arduino Mega, which acts as the central processing unit of the node. Optical sensors (UV, IR, and RGB), as well as physicochemical sensors such as pH, temperature, and turbidity, are interfaced through analog and digital input pins according to their signal requirements. A local display and LED indicators are connected to the node to provide real-time status information and visual feedback during operation. The detailed wiring and interconnections among the sensors, display, LED indicators, and the processing unit are illustrated in Figure 2.

#### 3.1.2. Gateway

The receiver is responsible for processing and storing the data transmitted from the sensing nodes in the designed architecture. In the current experimental phase, it receives data from the implemented sensing node. The gateway includes a Heltec WiFi LoRa 868 V2 module for receiving the LoRa data, an Arduino GIGA for processing the incoming data, and an SD card module equipped with a 32 GB SD card for local data storage. After the initial data storage stage, the collected data are transferred to a Raspberry Pi for machine learning training and model evaluation. The Raspberry Pi is connected to the Arduino GIGA through a USB connection and does not communicate directly with the Heltec module. This configuration supports the analysis and reconstruction of missing or invalid UV measurements.

Additionally, the system is designed to restart and resume data reception, processing, and storage even after power outages or temporary malfunctions, which improves reliability and operational continuity. As shown in Figure 2, the schematic of the gateway configuration highlights its role in facilitating communication between the sensing nodes and the processing unit.

### 3.2. Communication Details

LoRa technology has gained prominence as a wireless communication technology for wireless sensor networks, providing long-range data transmission with low power consumption. The modulation technique used by LoRa, known as Chirp Spread Spectrum (CSS), offers strong resistance to interference, making it suitable for harsh and noisy environments [32]. In addition, LoRa’s capability to decode signals below the noise floor supports reliable communication over long distances, even in densely populated IoT scenarios [33]. Therefore, integrating wireless sensor networks with LoRa technology presents a promising approach for monitoring pollutants in urban water systems [34]. This integration can improve the efficiency of data collection and enhance the responsiveness and adaptability of environmental monitoring systems, providing valuable insights into urban water management and pollution control [35].

Figure 3 presents the physical implementation of the proposed gateway. The main components are labeled as follows: (1) Arduino GIGA, (2) Arduino GIGA external Wi-Fi antenna, (3) Heltec LoRa WiFi 868 V2 antenna, (4) Heltec WiFi LoRa 868 V2 module, (5) integrated OLED display, (6) 3D-printed gateway enclosure, and (7) USB Type-C power cable. The compact enclosure measures approximately 145 mm × 60 mm × 40 mm, providing a portable housing for the communication and processing components.

Figure 4, illustrates the computer-aided design (CAD) of the proposed gateway enclosure developed for the central control unit. The enclosure was designed to provide mechanical protection for the electronic components while maintaining easy access to the communication interfaces, antennas, and power connection. The modular structure facilitates assembly, maintenance, and replacement of internal hardware components when required.

The main mechanical features are labeled as follows: (1) main enclosure body, (2) LoRa antenna mounting hole, (3) USB Type-C cable access port, (4) lid screw mounting posts, (5) enclosure fastening holes, (6) removable enclosure lid, (7) OLED display viewing window, and (8) internal retention clips for securing the Heltec WiFi LoRa 868 V2 module.

The communication architecture of the designed system ensures a continuous flow of data between the sensing nodes and the central receiver. In the current implementation phase, this communication flow is experimentally validated using the implemented sensing node. Each sensing node is equipped with a Heltec WiFi LoRa 868 V2 module, which transmits the collected data through the 868 MHz frequency band. Data transfer is performed through a Universal Asynchronous Receiver–Transmitter (UART) interface, where the Arduino Mega processes the sensor readings and forwards them to the Heltec module through the RX and TX pins. This configuration supports long-range data transmission in the implemented urban monitoring environment. This system architecture takes advantage of LoRa’s long-range communication capabilities while minimizing power consumption, which is essential for sustainable sensing operations in remote or difficult-to-access locations. In addition, the UART interface between the Arduino Mega and the Heltec module offers a straightforward and dependable mechanism for data handling, which is important for preserving data integrity in environmental monitoring applications [36].

In the implemented system, point-to-point LoRa communication was configured at a carrier frequency of 868 MHz. Based on the LoRa library configuration used in the implementation, the radio operated with a spreading factor (SF) of 11, a signal bandwidth of 125 kHz, a coding rate of 4/5, and a transmission power of 14 dBm using the PA_BOOST output. These parameters were used for communication between the sensing node and the gateway. The system employed direct LoRa communication and did not implement the LoRaWAN protocol.

On the receiving side, the central receiver, also equipped with a Heltec WiFi LoRa 868 V2 module, receives the transmitted data. These data are then forwarded to an Arduino GIGA through the RX and TX pins. The Arduino GIGA processes the incoming packets and stores them on an SD card for further analysis, after which the raw data are transmitted to a Raspberry Pi via USB for preprocessing and machine learning analysis. The receiver architecture ensures structured data storage and facilitates subsequent UV reconstruction tasks. The communication modules are configured to support low-power long-range data transmission. The integration of LoRa technology enables significant communication coverage, making it suitable for urban water monitoring deployments [37]. In addition, real-time transmission status is supported by displaying the transmitted data on the OLED screens of the Heltec modules, In addition, the transmitted and received data can be displayed on the OLED screens of the Heltec modules, providing real-time visual feedback during system operation.

Figure 5 illustrates the main steps of data collection and processing in the proposed sensing framework. Sensor data are first collected from the implemented node and transmitted to the gateway. The received data are then transferred to the Raspberry Pi for machine learning processing. The received sensor fields are then checked. When the UV measurement is available and valid, the record is stored for subsequent analysis. However, when the UV measurement is missing or invalid, the availability of the corresponding RGB and IR measurements is checked. If these measurements are available, the proposed prediction approach is applied to reconstruct the unavailable UV value. This workflow supports data continuity by reconstructing individual missing or invalid UV measurements within partially received records.

### 3.3. Deployment Environment

The proposed system was designed as a scalable five-node sensing framework for urban water monitoring in Gandia, specifically targeting underground urban water channels where surface water is collected and transported. The planned deployment considered five representative sensing locations distributed across the coastal area and the city center. In the current experimental validation, the implemented sensing node was positioned approximately two kilometers from the central gateway to evaluate long-range LoRa communication under realistic urban conditions.

In the current experimental phase, one sensing node was physically deployed and validated in the monitored urban water stream. The implemented node was installed in a floating position within the water flow. Water from the monitored stream naturally entered and passed through the sensing section of the node without manual introduction or replacement of water samples. This deployment strategy allowed the sensing system to operate directly with the monitored urban water while preserving the scalability of the overall five-node design.

In the optical sensing section, water from the monitored stream passed naturally through a glass tube integrated into the sensing unit. The optical transmitter and receiver components were positioned on opposite sides of the glass tube, allowing UV, IR, and RGB measurements to be obtained as the water passed through the sensing section. Other sensing parameters, including turbidity, temperature, and pH, were also monitored by the implemented node. This setup enabled continuous monitoring of physical and optical water parameters under the evaluated urban conditions. The central receiver was positioned approximately two kilometers from the implemented sensing node. This node-to-gateway distance was used to evaluate LoRa communication under the urban conditions of the experimental deployment, including the presence of buildings and infrastructure obstacles.

Figure 6 shows the overall communication configuration of the proposed LoRa-based sensing framework, consisting of the planned five-node architecture and the central gateway. The framework is designed to collect and transmit environmental data from the distributed sensing nodes to the gateway using point-to-point LoRa communication. The gateway then forwards the received data for storage, preprocessing, and machine learning analysis.

### 3.4. Sensor Baseline Evaluation and Repeatability

The sensors were evaluated using distilled water as a baseline reference under controlled conditions. This procedure was used to assess the stability and repeatability of the sensor responses rather than to establish absolute measurement accuracy. Distilled water, characterized by negligible turbidity and near-neutral pH, was used as the reference medium to establish baseline measurements for the sensing units. To minimize interference from external light sources, the optical sensors were physically designed to operate inside individual black plastic enclosures. This structural design prevented overlap and disturbance from ambient light, ensuring that each receiver detected only its corresponding light source. The internally emitted optical signals were also confined within their dedicated enclosures to improve signal isolation and reduce optical interference.

The UV, IR, and RGB sensors were specifically protected by placing their receivers in separate light-proof compartments to reduce optical interference and improve measurement stability. After assembling the sensor modules, their responses were evaluated under controlled measurement conditions to verify stable and repeatable sensor outputs. This strategy, combining optical isolation with controlled sensor evaluation, improved measurement stability and repeatability throughout the experiments. Since no certified reference instruments or multi-point calibration standards were used in this study, the reported results characterize sensor stability and repeatability under the evaluated conditions rather than absolute measurement accuracy.

### 3.5. Data Collection

Four separate measurement sessions were conducted on different days using the implemented sensing node deployed in the monitored urban water stream. During each day, data were collected between 9:00 AM and 5:00 PM at 15 min intervals, corresponding to four measurement points per hour. Across the four measurement sessions, 128 measurement points were considered. At each measurement point, multiple consecutive sensor readings were recorded, with the number of readings varying slightly between measurement points, averaging approximately 11.3 readings per point. This acquisition procedure resulted in a total of 1443 sensor records. Each record represents one set of sensor measurements acquired by the sensing node and stored for subsequent analysis.

Sensor measurements were acquired automatically by the Arduino according to the programmed acquisition procedure. Before each experimental session, the pH sensor was calibrated using a standard pH 7 reference solution. For each pH measurement, ten consecutive analog readings were acquired and sorted. The two highest and two lowest values were discarded, and the remaining six readings were averaged. The averaged reading was then converted to voltage, and the pH value was calculated using pH = −5.70V + 26.51, where V represents the voltage obtained from the averaged analog reading. The coefficient −5.70 represents the voltage-to-pH conversion slope used in the implemented sensor algorithm, while 26.51 is the calibration offset used in the implemented code. During each acquisition cycle, the connected sensors were read automatically, and the resulting measurements were prepared for transmission through the LoRa communication module. Water was not manually introduced or replaced during the experiments. Instead, water from the monitored urban stream naturally entered the sensing section, passed through the glass tube used for the optical measurements, and then exited the sensing section. The optical transmitter and receiver components were positioned on opposite sides of the glass tube to acquire the corresponding optical measurements as the water passed through the sensing section.

During data transmission, some received records were incomplete or contained malformed individual sensor fields, while the remaining sensor measurements were available. Missing or corrupted fields were observed for different sensing parameters and did not necessarily correspond to the loss of an entire record. For the UV reconstruction analysis, only records in which the UV measurement was missing or invalid while the corresponding RGB and IR measurements remained available were considered. This selection was made because the objective of the reconstruction model was specifically to estimate unavailable UV measurements using the available correlated optical measurements. Consequently, 75 UV measurements were identified for reconstruction, corresponding to approximately 5.2% of the 1443 collected records. These cases represent missing or invalid UV fields within partially received records rather than 75 completely lost LoRa packets.

For the experimental analysis, the dataset containing 1443 records was considered to assess measurement stability, sensor repeatability, and reconstruction performance. Statistical indicators, including the mean, minimum, maximum, and standard deviation, were calculated for each sensing parameter. In addition, the relative standard deviation (standard deviation as a percentage of the mean) was used to quantify data dispersion and evaluate the repeatability of the sensing platform.

### 3.6. Code Implementation

The code implementation in this project is organized into five main functional parts, each playing a crucial role in the overall operation of the proposed sensing framework. The software architecture was designed to support the scalable five-node deployment, while the current experimental validation was performed using the implemented sensing node.

The first two functional parts are related to the sensing node. They include sensor data acquisition through the Arduino Mega and the wireless transmission of the processed measurements through the Heltec LoRa module. The next two parts correspond to the gateway, including packet reception through the Heltec LoRa receiver and structured data storage on the SD card through the Arduino GIGA. The final functional part implements the machine learning workflow, where missing or invalid UV measurements are reconstructed using the proposed predictive models. This modular structure allows the sensing, communication, storage, and data-reconstruction functions to be clearly separated within the overall system architecture.

#### 3.6.1. Sensing Node Firmware

The Arduino Mega firmware is responsible for collecting data from the UV, IR, RGB, turbidity, temperature, and pH sensors. During system setup, serial communication, LCD visualization, and all sensing modules are initialized to ensure stable operation. Raw sensor acquisition includes RGB optical channels, IR, turbidity, pH, and temperature measurements. For analog sensors, ten consecutive samples are acquired, sorted, and averaged using the middle values to suppress impulsive noise and improve robustness before wireless transmission. For the DS18B20 temperature sensor, a direct digital reading is used. The sensor data acquisition and local processing procedure is summarized in Algorithm 1, including sensor sampling, signal processing, conversion of the acquired values, local display, and preparation of the structured transmission frame.

**Algorithm 1.** Sensor Data Acquisition and Local ProcessingInput: RGB, UV, IR, turbidity, pH, temperature Output: Processed sensing values ready for transmission1.  Acquire 10 consecutive samples for each analog sensor2.  Sort the samples3.  Compute the average of the middle six values4.  Convert the averaged values according to the implemented sensor processing procedures5.  Calculate the pH value using the implemented voltage-to-pH conversion equation after calibration with a standard pH 7 reference solution6.  Convert turbidity to NTU7.  Convert UV and IR to voltage levels8.  Read digital temperature value9.  Display the processed data locally on LCD10.Package all measurements into a structured transmission frame

The processed sensor data are then formatted into a transmission string and forwarded to the Heltec sender module. The main loop repeats the acquisition, filtering, visualization, and transmission cycle according to the programmed acquisition procedure.

#### 3.6.2. LoRa Communication Modules

The Heltec WiFi LoRa 868 V2 modules are responsible for wireless communication between the sensing node and the gateway. Within the scalable five-node architecture, each sender is associated with one sensing node, while the current experimental validation was performed using the implemented node. During initialization, serial communication and the LoRa modules are configured to operate at 868 MHz for long-range data transmission. The sender encapsulates the incoming sensor measurements together with a node identifier into LoRa packets. On the gateway side, the Heltec receiver continuously listens for incoming packets, parses the received payloads, and forwards them through serial communication to the Arduino GIGA. A local OLED display on both the sender and receiver modules provides basic visual feedback on the transmitted and received data during system operation.

#### 3.6.3. Gateway Storage and Data Forwarding

The Arduino GIGA with SD card module acts as the central storage unit of the proposed framework. Incoming packets received from the Heltec gateway are parsed, source-tagged, and stored in node-specific files on the microSD card to support organized logging in the scalable five-node architecture. During initialization, serial communication and SD card access are configured to support local data storage. If initialization fails, the system halts to avoid invalid data logging. The stored raw data are subsequently forwarded to the Raspberry Pi for preprocessing, machine learning training, and missing UV reconstruction.

### 3.7. Analyzing in Raspbery Pi

The Raspberry Pi acts as the edge-level processing unit of the proposed monitoring framework. After raw sensor data are received from the gateway and stored on the SD card, the records are transferred to the Raspberry Pi for preprocessing, model execution, and missing UV reconstruction. At the beginning of the workflow, the required Python 3.12 libraries for data handling, preprocessing, statistical modeling, and machine learning execution are imported. The stored Comma-Separated Values (CSV) records are then loaded into a structured data frame for analysis. The preprocessing stage includes conversion of invalid UV entries into Not a Number (NaN), median imputation of missing R, G, B, and IR values, and preparation of the final feature matrix consisting of R, G, B, and IR measurements. These variables serve as the predictive inputs for UV reconstruction.

For nonlinear relationship modeling, polynomial feature expansion (degree = 2) is first generated to support the baseline polynomial regression workflow. In addition, the same preprocessed dataset is passed to the broader machine learning pipeline, where advanced models including SVR, KNN, Random Forest, and MLP are evaluated and compared. For the initial model evaluation, the dataset is divided into training and testing subsets using an 80/20 random split. In addition, Leave-One-Session-Out (LOSO) validation is performed to assess model generalization across different experimental sessions. During model execution, the missing UV entries are identified, their corresponding RGB and IR features are extracted, and the selected trained model is used to estimate the missing UV values. Based on the initial random train–test evaluation presented in Section 4, the Random Forest model achieved the highest R^2^ (0.991) and was therefore selected for the missing UV reconstruction implemented in the Raspberry Pi workflow.

Finally, the completed dataset, including the reconstructed UV values, is stored both in the Raspberry Pi local storage and the external USB backup directory. This edge-processing workflow enables local intelligence, supports data continuity, and improves the overall robustness of the monitoring system.

### 3.8. Machine Learning

This section presents a machine-learning framework to reconstruct missing or invalid UV measurements in the proposed sensing platform. In the collected data, some records contained missing or invalid UV measurements while the corresponding R, G, B, and IR measurements remained available. The proposed edge-computing approach uses these available optical measurements to estimate the unavailable UV values, applying sensor-based dynamics to full-state estimation principles [38].

To ensure alignment with a complete machine-learning workflow, both baseline regression models and more advanced ML regressors were evaluated. Multiple Linear Regression and second-degree Polynomial Regression were used as reference baselines, while Support Vector Regression (SVR), k-Nearest Neighbors (KNN), Random Forest (RF), and a Neural Network (MLP) were implemented to capture nonlinear relationships and interactions between optical signals and UV intensity. Model performance was assessed using standard regression metrics (MAE, RMSE, and R^2^), allowing a transparent comparison across methods.

#### 3.8.1. Data Preprocessing

Before training any predictive model, the dataset was preprocessed to ensure consistency and avoid bias introduced by missing or corrupted values. The preprocessing focused on retaining the relevant predictors for UV reconstruction (R, G, B, IR), handling formatting issues, and removing incomplete records that could degrade model training. A preliminary correlation screening was used as a simple filter to confirm that the optical channels are the most informative predictors for UV estimation. Non-relevant columns were excluded from the modeling input to keep the pipeline efficient and reproducible.

Missing or invalid numeric entries were converted to NaN and imputed using robust statistics. Median imputation was applied to R, G, B, and IR to reduce the influence of outliers, while missing UV values were intentionally kept as NaN during training set preparation so that the model was trained only on valid UV labels. After correction and imputation, any remaining incomplete rows in the selected modeling columns were removed to produce a clean dataset for model development. The data preprocessing procedure used to prepare the sensor measurements for model development is summarized in Algorithm 2, including data selection, numeric conversion, handling of invalid entries, treatment of missing predictor values, and preservation of missing UV measurements for subsequent reconstruction.

**Algorithm 2.** Data Preprocessing PipelineInput: Raw sensor dataset Output: Cleaned dataset for model developmentLoad the raw CSV dataset into a data frameSelect the required columns: UV, R, G, B, IRConvert all selected columns into numeric formatReplace invalid entries with NaNApply median imputation to R, G, B, and IRPreserve missing UV entries for later reconstructionRemove incomplete rows from the training subsetReturn the cleaned dataset

#### 3.8.2. Exploratory Data Analysis (EDA)

Before model training, an exploratory analysis was performed to understand dataset variability and inspect how optical measurements relate to UV intensity. This step verifies that the dataset contains sufficient variation for learning and provides a first indication of which predictors are most informative. Correlation analysis. Pearson correlation coefficients were computed to examine linear dependencies among R, G, B, IR, and UV. The correlation matrix was visualized as a heatmap (Figure 6), providing a quick overview of pairwise relationships and potential multicollinearity among predictors. Descriptive statistics. Summary statistics including mean, standard deviation, minimum, maximum, and quartiles were computed for the selected columns to characterize the operating range of each measurement channel. This confirmed that UV values span a meaningful range and further justified the use of nonlinear learning models. The exploratory data analysis procedure is summarized in Algorithm 3, including descriptive statistical analysis, Pearson correlation analysis, correlation-matrix generation, and examination of the relationships among the optical sensor variables.

**Algorithm 3.** Exploratory Data AnalysisInput: Cleaned dataset Output: Statistical characterization and correlation matrixCompute descriptive statistics for UV, R, G, B, IREstimate Pearson correlation coefficients among all variablesGenerate the correlation matrixVisualize the matrix as a heatmapAnalyze variable ranges and pairwise relationships

#### 3.8.3. Model Development and Training

Model Development, Training, and Selection: After preprocessing and exploratory analysis, several regression and machine learning models were implemented to estimate UV intensity from optical sensor measurements. The objective was to compare classical regression approaches with more advanced machine learning techniques in terms of prediction accuracy and generalization capability.

For the initial random train–test evaluation, the dataset was divided into training and testing subsets using an 80/20 split. In addition, Leave-One-Session-Out (LOSO) validation was performed to evaluate cross-session generalization. To avoid scale-related bias in distance-based and kernel-based models, feature normalization was applied using standardization (zero mean and unit variance) before training SVR, KNN, and MLP models.

##### Implemented Models

- Multiple Linear Regression (baseline)
- Polynomial Regression (degree = 2)
- Support Vector Regression (SVR)
- k-Nearest Neighbors (KNN)
- Random Forest Regressor (RF)
- Multi-Layer Perceptron (MLP)

The session-aware model training and evaluation workflow is summarized in Algorithm 4. The same Leave-One-Session-Out (LOSO) validation procedure was applied to all evaluated models to provide a consistent assessment of cross-session generalization.

**Algorithm 4.** Model Training and Evaluation WorkflowInput: Cleaned feature matrix X = [R, G, B, IR], target vector y = UV, session labels Output: Cross-session performance of the evaluated predictive models1.  Organize the dataset according to the four experimental sessions2.  Define Leave-One-Session-Out (LOSO) cross-validation3.  For each LOSO fold, use three sessions for training and one complete session for testing4.  Standardize the feature space for SVR, KNN, and MLP using the training data5.  Train the Multiple Linear Regression model6.  Generate second-degree polynomial features and train the Polynomial Regression model7.  Train the SVR model using the RBF kernel8.  Train the KNN regressor (k = 5)9.  Train the Random Forest regressor (300 trees)10.Train the MLP neural network11.Evaluate each model using MAE, RMSE, and R^2^ on the held-out session12.Repeat the procedure until each session has served once as the test session13.Report MAE, RMSE, and R^2^ as mean ± standard deviation across the four LOSO folds

The performance of all models was compared using MAE, RMSE, and R^2^. While linear regression provides a simple baseline, the nonlinear models were evaluated to investigate their ability to capture more complex relationships between the optical measurements and UV values.

In the initial random train–test evaluation, Random Forest achieved the highest predictive performance among the evaluated models. However, the session-aware LOSO evaluation showed considerably greater performance variability across independent experimental sessions. Therefore, the random train–test results should be interpreted as within-dataset performance and not as evidence of consistently strong generalization across different measurement sessions.

## 4. Data Analysis and Model Evaluation

This section presents the predictive modeling results, cross-session generalization analysis, and energy implications of the proposed UV reconstruction framework. Both classical regression and advanced machine learning approaches were examined to determine the most suitable model for estimating missing UV measurements from optical sensor readings (R, G, B, IR).

### 4.1. Multiple Linear Regression (Baseline Model)

Multiple Linear Regression (MLR) was first implemented as a baseline model to establish a reference performance level. The relationship between UV intensity and the optical sensor measurements was modeled as:

(1) $$UV\;=\;\beta_{0}+\;\beta_{1}R\;+\;\beta_{2}G\;+\;\beta_{3}B\;+\;\beta_{4}IR\;+\;\varepsilon$$

where *β0* represents the intercept, *β1*–*β4* are the regression coefficients, and *ε* epsilon *ε* denotes the random error term. As shown in Equation (1), the baseline linear model assumes that UV intensity can be represented as a weighted linear combination of the optical measurements *R*, *G*, *B*, and *IR*. Although computationally efficient and easily interpretable, this model assumes a purely linear relationship between the predictors and UV intensity. In the initial random train–test evaluation, the obtained R^2^ of 0.12 indicates that the linear model explained only a limited proportion of the variance in UV intensity, suggesting that a purely linear model was insufficient to represent the relationships in the evaluated dataset.

### 4.2. Polynomial Regression (Second-Order)

To capture nonlinear dependencies, a second-degree polynomial regression model was implemented by expanding the feature space to include quadratic and interaction terms [39]:

(2) $$\begin{array}{c} UV\;=\;\beta_{0}+\;\beta_{1}R\;+\;\beta_{2}G\;+\;\beta_{3}B\;+\;\beta_{4}IR\;+\;\beta_{5}R^{2}+\;\beta_{6}G^{2}+\;\beta_{7}B^{2}+\;\beta_{8}IR^{2}+\;\beta_{9}RG\;+\\ \;\beta_{\left\{10\right\}RB}+\;\beta_{\left\{11\right\}RIR}+\;\beta_{\left\{12\right\}GB}+\;\beta_{\left\{13\right\}GIR}+\;\beta_{\left\{14\right\}BIR}+\;\epsilon \end{array}$$

As shown in Equation (2), the inclusion of quadratic and interaction terms allows the model to capture nonlinear dependencies among optical sensor measurements. This model significantly improved predictive capability, achieving *R*^2^ = 0.9633, which explains approximately 96.3% of the variance in UV intensity. The substantial improvement confirms that nonlinear interactions among optical measurements play a critical role in UV estimation. This result demonstrates that feature interactions and quadratic effects are essential for reliable *UV* reconstruction.

However, polynomial regression increases computational complexity and may introduce overfitting if the polynomial degree is not carefully controlled, a limitation that is also a known challenge in more complex deep polynomial neural networks [40]. This observation motivated the evaluation of additional machine learning regressors in the next subsection.

### 4.3. Comparative Machine Learning Performance

To comprehensively evaluate the predictive capability of different modeling strategies, several regression and machine learning algorithms were implemented and compared. The objective was to determine which approach most effectively captures the relationship between optical sensor measurements (R, G, B, IR) and UV intensity. Prior to model development, the statistical characteristics and distributions of the processed sensor parameters were examined, as illustrated in Figure 7. The histogram and Kernel Density Estimation (KDE) curves reveal that the distributions of the optical channels are neither perfectly linear nor strictly Gaussian. These patterns suggest the presence of nonlinear interactions among features, thereby motivating the use of nonlinear machine learning models in addition to classical regression. The initial predictive performance of all evaluated models is summarized in Table 1, where Mean Squared Error (MSE) and the coefficient of determination (R^2^) are reported using the same random train–test evaluation procedure. A more conservative session-aware Leave-One-Session-Out (LOSO) validation is subsequently presented in Section 4.4.

As shown in Table 1, the baseline Multiple Linear Regression model exhibits limited explanatory capability (R^2^ = 0.12), confirming that the relationship between optical signals and UV intensity cannot be adequately described using a purely linear formulation. Polynomial regression significantly improves the predictive performance (R^2^ = 0.9633), demonstrating that incorporating second-order and interaction terms partially captures the nonlinear behavior observed in Figure 7. However, although polynomial expansion increases model flexibility, it remains a parametric method with limited adaptability to complex feature interactions.

Among the evaluated models, Random Forest achieved the best performance in the initial random train–test evaluation, with the lowest MSE (0.00022) and the highest coefficient of determination (R^2^ = 0.991). This result suggests that Random Forest was able to capture the nonlinear relationships between the RGB and IR measurements and the corresponding UV values within this evaluation. The MLP and SVR models also achieved high R^2^ values (R^2^ > 0.97), while KNN showed slightly lower performance. However, these results are based on the random train–test split and should be interpreted together with the session-aware LOSO validation presented in Section 4.4, which provides a more conservative assessment of model generalization across experimental sessions.

Overall, the random train–test results indicate that nonlinear models can capture the relationship between the available optical measurements and UV values within the collected dataset. While Polynomial Regression substantially improved performance over Linear Regression, Random Forest achieved the highest predictive performance in the random train–test evaluation. However, the LOSO results showed considerable performance variation between experimental sessions. Therefore, the random train–test results should be interpreted together with the session-aware validation results when assessing the reconstruction performance of the proposed approach.

For reproducibility, fixed model configurations were used for all evaluated methods. Multiple Linear Regression was implemented using ordinary least-squares regression without additional hyperparameter tuning. Polynomial Regression used second-order polynomial features followed by linear regression. Support Vector Regression (SVR) employed a radial basis function (RBF) kernel with C = 1.0, epsilon = 0.1, and gamma = ‘scale’. K-Nearest Neighbors (KNN) regression was configured with k = 5 and uniform weighting. Random Forest regression used 300 decision trees, with no predefined maximum tree depth, a minimum of two samples required to split an internal node, a minimum of one sample per leaf, and a random seed of 42. The MLP neural network consisted of two hidden layers with 64 and 32 neurons, respectively, using ReLU activation and the Adam optimizer, with a learning rate of 0.001, a maximum of 2000 iterations, and a random seed of 42. Input features were standardized before training the SVR, KNN, and MLP models. The same Leave-One-Session-Out (LOSO) validation procedure was applied to all models.

### 4.4. Model Validation and Generalization

Since the experimental setup involved a single sensing node, spatial validation across multiple deployment locations was not applicable. To reduce the risk of information leakage associated with repeated measurements collected within the same experimental session, model generalization across experimental sessions was evaluated using Leave-One-Session-Out (LOSO) cross-validation. The dataset was organized into four experimental sessions according to the data-acquisition sequence. Four validation folds were performed, with one complete session used exclusively for testing in each fold and the remaining three sessions used for model training. Records with missing UV measurements were excluded from the performance evaluation because no reference UV value was available for calculating the prediction error.

The same session-aware validation procedure was applied to all evaluated regression models to provide a consistent comparison. Model performance was assessed using the Mean Absolute Error (MAE), Root Mean Squared Error (RMSE), and coefficient of determination (R^2^). The results are reported as the mean ± standard deviation across the four held-out sessions. Table 2 summarizes the LOSO validation results for all evaluated models in terms of MAE, RMSE, and R^2^.

The session-aware validation produced substantially greater variability than random sample-level validation. Among the evaluated models, KNN achieved the lowest mean MAE and RMSE, followed closely by Random Forest. However, the negative mean R^2^ values indicate limited generalization across some held-out experimental sessions. This suggests that the relationships between the optical measurements varied across sessions and that performance estimates obtained using random sample-level splitting may be overly optimistic. These findings highlight the importance of session-aware validation and indicate that a larger multi-session dataset and further evaluation under independent measurement conditions are required to more comprehensively assess model generalization.

### 4.5. Overall Analysis of Sensors

The sensing parameters exhibit substantially different numerical ranges because they represent different physical quantities and sensor output scales. Therefore, direct comparison of their absolute mean values is not appropriate. To provide a scale-independent assessment of measurement stability, the relative standard deviation (RSD) was used to evaluate the variability of repeated measurements. The results indicate low relative variability across the evaluated sensing parameters under the controlled measurement conditions.

Although the RGB and IR measurements exhibit larger numerical values than UV, temperature, pH, and turbidity, their relative dispersion remains small. This indicates that the observed differences in magnitude are primarily associated with the different sensor output ranges rather than differences in measurement stability. These results characterize the repeatability of the sensing system under the evaluated conditions; they should not be interpreted as a direct assessment of absolute measurement accuracy, since certified reference instruments and multi-point calibration standards were not used in this study.

Figure 8a presents the normalized mean values of the optical sensor measurements, including UV, R, G, B, and IR. Normalization was applied to enable a meaningful visual comparison among sensor outputs with substantially different numerical ranges. The figure shows the relative magnitude of the normalized responses and should not be interpreted as a direct comparison of the sensors’ absolute measurement accuracy. Figure 8b further presents the relative variability of the sensor responses, providing a scale-independent assessment of measurement repeatability. Together, these results provide an experimental characterization of sensor stability under the evaluated conditions and support the subsequent data preprocessing and machine learning analysis.

### 4.6. Energy Consumption

Based on the specifications reported in the manufacturers’ datasheets, an estimated power budget for the proposed sensing platform was derived. Table 3 presents the typical power consumption of the main hardware components under nominal operating conditions. This estimation provides a general overview of the nominal energy requirements of the proposed system. It should be noted that actual power consumption may vary depending on the processing load, sensing frequency, and wireless communication activity. In addition, higher-power devices such as the Raspberry Pi operate at the gateway level and are not part of the distributed sensing node itself. Overall, the moderate energy requirements support the suitability of the system for deployment in resource-constrained and remote environments.

#### 4.6.1. Total Energy and Time Consumption for Node 1

The estimated power consumption of Node 1 was evaluated based on the nominal power requirements of its hardware components. For the acquisition-related energy analysis, an active duration of approximately 10 s was considered for each measurement cycle. The energy required for one complete acquisition cycle was estimated using the nominal active power consumption of the node. The active power of Node 1 was estimated as $P_{node}=2.995$ W based on the nominal power requirements of its hardware components. Based on an active power of 2.995 W and an active duration of $t_{active}=10$ s, the estimated energy consumption of one complete acquisition cycle ($E_{cycle}$) is calculated using Equation (3):

(3) $$E_{cycle}=P_{node}\times \frac{t_{active}}{3600}$$

Substituting the specific values yields Equation (4):

(4) $$E_{cycle}=2.995\times \frac{10}{3600}=0.00832\;\mathrm{Wh}$$

Accordingly, the estimated energy consumption of one complete acquisition cycle is 0.00832 Wh. Among the 1443 collected records, 75 UV measurements (approximately 5.2%) were missing or invalid and were reconstructed using the proposed machine learning approach.

To estimate the potential energy benefit of the reconstruction approach, it was assumed that, in the absence of reconstruction, each missing UV measurement would require one additional complete 10 s acquisition cycle. Since 75 UV measurements were reconstructed, the corresponding avoided energy consumption ($E_{saved}$) was estimated using Equation (5):

(5) $$E_{saved}=N_{reconstructed}\times E_{cycle}$$

Substituting the specific values yields Equation (6):

(6) $$E_{saved}=75\times 0.00832=0.624\;\mathrm{Wh}$$

Thus, under this model-based reacquisition scenario, reconstructing the 75 missing UV measurements potentially avoids approximately 0.624 Wh of node energy consumption.

Without the reconstruction approach, the 75 missing UV measurements would correspond to 75 additional acquisition cycles under the assumed reacquisition scenario. Therefore, the total number of acquisition cycles would increase from 1443 to 1518. The estimated reduction in acquisition-related energy consumption can therefore be expressed using Equation (7):

(7) $$\mathrm{Energy}\;\mathrm{Reduction}\;(\%)=\frac{N_{avoided}}{N_{original}+N_{avoided}}\times 100$$

Substituting the specific values yields Equation (8):

(8) $$\mathrm{Energy}\;\mathrm{Reduction}=\frac{75}{1443+75}\times 100=4.94\%$$

Accordingly, the proposed reconstruction approach corresponds to an estimated 4.94% reduction in acquisition-related energy consumption under the assumed one-reacquisition-per-missing-UV scenario.

For the 10,000 mAh battery considered in this study, a nominal battery voltage of 3.7 V corresponds to a nominal energy capacity ($E_{battery}$) of 37 Wh, calculated via Equation (9):

(9) $$E_{battery}=C_{battery}\times V_{battery}$$

Substituting the specific values yields Equation (10):

(10) $$E_{battery}=10\;\mathrm{Ah}\times 3.7\;V=37\;\mathrm{Wh}$$

The estimated 0.624 Wh of avoided reacquisition energy therefore corresponds to approximately 1.69% of the nominal battery energy capacity, as shown in Equation (11):

(11) $$\frac{0.624}{37}\times 100=1.69\%$$

This value represents a model-based estimate relative to the nominal battery capacity and should not be interpreted as a directly measured extension of battery lifetime.

Node 1 consists of the following components:

- Arduino Mega
- pH sensor
- IR sensor
- UV sensor
- RGB sensor
- Turbidity sensor
- LCD (4 cm × 8 cm)
- IR receiver
- RGB receiver
- UV receiver
- Heltec LoRa WiFi 868 V2

Table 4 summarizes the estimated energy consumption of Node 1 and the potential acquisition-related energy saving under the assumptions described above.

#### 4.6.2. Total Energy Consumption for Gateway

The energy consumption of the Gateway was estimated based on the nominal power requirements of its hardware components. The Gateway consists of:

Arduino GIGA
Heltec LoRa WiFi 868 V2
SD Module
Raspberry Pi

Table 5 presents the estimated energy consumption of the Gateway based on the nominal power requirements of these hardware components. The estimated total active power consumption of the Gateway is 15.32 W. Unlike the sensing-node analysis, no fixed percentage reduction was applied to the Gateway power consumption as a consequence of the machine learning reconstruction. The reconstruction of missing UV measurements potentially avoids additional acquisition cycles at the sensing-node level; therefore, directly applying the 5.2% missing-data rate to the total Gateway power consumption would not provide a physically justified estimate of energy saving. Accordingly, Table 5 reports the Gateway energy consumption as a baseline estimate without attributing a fixed energy reduction to the machine learning reconstruction.

#### 4.6.3. Total Energy Consumption for the Entire System

Table 6 presents the estimated energy consumption of the complete system, including the sensing-node architecture and the Gateway, based on the nominal power requirements of the hardware components. The estimated total active power consumption of the complete system is 30.295 W.

Unlike the previous analysis, the 5.2% proportion of missing UV measurements was not directly applied as a reduction factor to the total system power consumption. This percentage represents the proportion of missing or invalid UV measurements in the collected dataset and does not imply an equivalent reduction in the power consumption of all system components. As described in Section 3.6.1, the potential energy benefit of the machine learning reconstruction was therefore estimated at the sensing-node acquisition level, where avoiding 75 additional 10 s reacquisition cycles corresponds to approximately 0.624 Wh of avoided energy and an estimated 4.94% reduction in acquisition-related energy consumption under the assumed reacquisition scenario.

Accordingly, Table 6 reports the baseline energy consumption of the complete system without applying a fixed machine-learning-based reduction to the total system power.

## 5. Results

The statistical analysis of the repeated measurements provides an assessment of the stability and repeatability of the sensing platform under the evaluated conditions. The percentage of standard deviation relative to the mean values remained low across all measured parameters, indicating minimal fluctuation during repeated measurements. These results indicate good repeatability of the sensing hardware under the evaluated conditions.

Table 7 summarizes the repeatability statistics of the measured parameters under controlled conditions, including mean, standard deviation, minimum, maximum, and relative standard deviation (RSD, %). The UV sensor recorded a mean value of 1.66 with a standard deviation of 0.028 (RSD = 1.699%), indicating low relative variability. Similarly, the RGB and IR sensors exhibited very low relative dispersion (below 0.2%), indicating stable optical responses during the repeated measurements. Temperature and pH measurements also showed minimal variability, while turbidity remained constant during the controlled experiments.

### 5.1. Machine Learning Reconstruction Performance

Beyond sensor stability, the main objective of this study was to reconstruct missing or invalid UV measurements using the available optical sensor data (R, G, B, and IR). Approximately 5.2% of the UV measurements in the dataset were missing or invalid and were reconstructed using the proposed approach. Multiple modeling approaches were evaluated to assess reconstruction performance. In the initial random train–test evaluation, the baseline Multiple Linear Regression model showed limited performance (R^2^ = 0.12), while second-order Polynomial Regression achieved a considerably higher R^2^ of 0.963.

To further evaluate the reconstruction capability, advanced machine learning models were implemented. In the initial random train–test evaluation, Random Forest achieved the highest performance among the evaluated models, with R^2^ = 0.991 and an MSE of 0.00022. However, the session-aware LOSO evaluation produced substantially greater variability between experimental sessions. Under LOSO validation, KNN achieved the lowest mean MAE and RMSE, followed closely by Random Forest, while the mean R^2^ values were negative for all evaluated models. These results indicate that the high performance obtained from the random train–test evaluation should be interpreted as within-dataset predictive performance and does not demonstrate consistent generalization across independent experimental sessions.

### 5.2. Energy Impact of ML-Based Reconstruction

The reconstruction of missing or invalid UV measurements may provide an energy benefit by avoiding additional acquisition cycles. In the collected dataset, 75 UV measurements were missing or invalid while the corresponding RGB and IR measurements remained available. Based on an estimated active power of 2.995 W and an acquisition duration of approximately 10 s, one complete acquisition cycle requires approximately 0.00832 Wh. Assuming that each unavailable UV measurement would otherwise require one additional complete acquisition cycle, reconstructing these 75 UV values could avoid approximately 0.624 Wh of acquisition energy. This corresponds to an estimated 4.94% reduction in acquisition-related energy consumption under the stated assumption. The implemented system did not automatically trigger an additional acquisition cycle when a UV measurement was missing or invalid. Therefore, the calculated 4.94% reduction represents a scenario-based estimate of potential acquisition-related energy saving rather than an experimentally measured energy reduction.

## 6. Discussion

The results show that machine learning can be used to reconstruct missing or invalid UV measurements from the available RGB and IR data. In the initial random train–test evaluation, Random Forest provided the best result, with an R^2^ of 0.991. However, when the models were evaluated using the more conservative Leave-One-Session-Out (LOSO) approach, their performance varied considerably between experimental sessions. KNN showed the lowest average MAE and RMSE, followed closely by Random Forest, but the negative mean R^2^ values indicate that none of the models generalized consistently across all sessions.

This difference between the two validation approaches is important. Measurements collected during the same experimental session are likely to share similar conditions and characteristics. Therefore, randomly distributing these measurements between the training and testing sets can make the prediction task easier and lead to more optimistic results. The LOSO evaluation provides a more conservative indication of how the models may behave when applied to data collected during a different session. The results also show that KNN and Random Forest achieved lower mean prediction errors than the linear and polynomial models under the LOSO evaluation. Nevertheless, the variation between sessions shows that UV reconstruction should be used as a supporting method when individual UV measurements are unavailable, rather than as a replacement for the physical UV sensor.

The reconstruction approach may also provide an energy benefit by avoiding additional measurement cycles when a UV value is missing or invalid. This does not mean that the machine learning model directly reduces the power consumption of the hardware. Instead, the potential saving comes from avoiding a complete reacquisition cycle when the missing UV value can be reconstructed from the available measurements. The actual effect on battery lifetime will depend on practical factors such as the sampling frequency, usable battery energy, power-conversion efficiency, and operating duty cycle. For this reason, further experiments involving more independent measurement sessions and longer field deployments are needed to evaluate both the generalization of the reconstruction models and their practical energy benefits.

### 6.1. Potential Acquisition-Related Energy Reduction

The energy analysis presented in Section 4 indicates that UV reconstruction may reduce acquisition-related energy consumption by avoiding additional measurement cycles when individual UV values are missing or invalid while the corresponding optical predictors remain available. As illustrated in Figure 9, the potential energy benefit is expressed in terms of the acquisition energy associated with the analyzed dataset rather than as a direct reduction in instantaneous system power. Under the stated reacquisition assumption, this benefit is model-based and should not be interpreted as a directly measured reduction in total system power consumption.

From a practical perspective, the potential energy benefit of the reconstruction approach depends on the frequency of missing measurements, the duration and power demand of each acquisition cycle, and the operating strategy adopted in a long-term deployment. Therefore, the estimated energy reduction reported in this study should be interpreted within the specific assumptions of the proposed energy model rather than as a fixed energy-saving rate applicable to all deployment conditions.

### 6.2. Operating Time

The actual operating lifetime of the sensing system depends on several factors, including usable battery energy, power-conversion efficiency, acquisition frequency, and the active/inactive duty cycle. Since these parameters were not fully characterized experimentally, a fixed battery operating time is not reported in this study. The energy analysis presented in Section 4 instead characterizes the acquisition-related energy requirements of the implemented sensing node under explicitly stated operating assumptions.

Avoiding unnecessary additional acquisition cycles may reduce acquisition-related energy consumption and potentially contribute to longer operating autonomy. However, the resulting extension in battery lifetime cannot be directly inferred from the estimated acquisition-energy reduction because it also depends on the usable battery energy, conversion losses, sampling schedule, and long-term duty cycle. Therefore, the present results should be interpreted as an assessment of potential acquisition-related energy savings rather than as a prediction of a fixed operating lifetime. Figure 10 summarizes the model-based relationship between acquisition-related energy consumption and the potential energy benefit of avoiding additional acquisition cycles.

#### Comparison with Previous Studies

Water quality monitoring and surveillance are commonly performed using data collected from dedicated sensing platforms and subsequently analyzed using statistical or machine learning methods [24,25,26,27,28]. Table 8 compares representative previous studies in terms of the models employed, input parameters, reported predictive performance, and practical implementation. Previous studies [13,18,21,24,25,26,27,28] have investigated different combinations of water-quality parameters and computational models; however, several of these studies primarily focused on computational analysis, with limited emphasis on physical implementation and experimental evaluation. In contrast, the present study combines multi-sensor measurements, point-to-point LoRa communication, machine-learning-based UV reconstruction, and the physical implementation and experimental evaluation of one sensing node.

In the initial random train–test evaluation, Random Forest achieved the highest coefficient of determination among the evaluated models (R^2^ = 0.991). However, the session-aware LOSO evaluation presented in Section 4.4 produced substantially greater variability across independent experimental sessions, indicating that the random sample-level evaluation provides a more optimistic estimate of predictive performance. Under LOSO validation, KNN achieved the lowest mean MAE and RMSE, while none of the evaluated models demonstrated consistently strong cross-session generalization. These findings emphasize the importance of session-aware validation and indicate that further evaluation using a larger number of independent acquisition sessions is required before strong generalization claims can be made.

### 6.3. Limitations

The proposed system demonstrated the feasibility of UV reconstruction under the evaluated conditions; however, several limitations should be considered.

First, although LoRa provides long-range communication, signal quality may degrade in dense urban environments due to physical obstructions and electromagnetic interference. Although the UV reconstruction algorithm can compensate for individual missing or invalid UV measurements when the corresponding optical predictors remain available, severe communication failures may still affect the overall system reliability.

Second, the current experimental evaluation was conducted using one sensing node deployed in the monitored urban water stream. Water naturally entered the sensing section, passed through the glass tube used for the optical measurements, and then exited the sensing section without manual introduction or replacement of prepared water samples. Therefore, the measurements represented the water conditions encountered during the evaluated deployment. However, the experiments were limited to one sensing node and four measurement sessions. Additional experiments with more sensing nodes, longer monitoring periods, and different water conditions are required to further evaluate the robustness and generalization of the proposed framework.

Third, the use of machine learning for UV reconstruction introduces an additional limitation. The proposed reconstruction approach is not intended to replace the physical UV sensor, which remains necessary for direct measurement and for capturing unexpected or anomalous changes in water conditions. The machine learning model is used only as a temporary data-recovery mechanism when an individual UV measurement is missing or invalid while the corresponding RGB and IR measurements remain available. Under unusual or out-of-distribution environmental conditions, direct UV measurements should be prioritized, since a model trained on previously observed conditions may not reliably reconstruct such unexpected events.

Fourth, the energy consumption values were estimated under nominal operating conditions and should therefore be interpreted as model-based estimates rather than directly measured energy savings. In long-term deployments, variations in sensing frequency, transmission intervals, duty cycle, and environmental factors may modify the actual energy consumption. In addition, the current prototype has not yet undergone extended deployment in harsh or fully submerged wastewater environments. For large-scale applications, further hardware ruggedization, waterproof encapsulation, and environmental protection would be necessary.

Fifth, the LCD display was included in the current prototype to facilitate local monitoring, debugging, and verification of sensor measurements during system development and experimental testing. However, the display is not essential for the normal operation of the sensing node. In a long-term battery-powered deployment, the LCD and its backlight can be disabled or removed to further reduce energy consumption.

Finally, the Arduino Mega was selected for the current prototype because of its sufficient number of input/output interfaces and ease of integration with multiple sensors. However, it is not optimized for ultra-low-power operation. Future versions of the sensing node could employ lower-power microcontrollers, such as ARM Cortex-M0/M3/M4-based platforms, together with appropriate sleep modes and power-management strategies to further reduce energy consumption during long-term deployments.

## 7. Conclusions

This study presented a LoRa-based water quality monitoring system that combines multiple sensors with machine learning to reconstruct missing or invalid UV measurements. The implemented sensing node included UV, IR, RGB, pH, turbidity, and temperature sensors, while RGB and IR measurements were used as inputs for UV reconstruction. A total of 1443 sensor records were collected during the experiments, including 75 records in which the UV value was missing or invalid while the corresponding RGB and IR measurements were available.

In the initial random train–test evaluation, Multiple Linear Regression showed limited performance (R^2^ = 0.12), while second-order Polynomial Regression achieved an R^2^ of 0.963. Among the machine learning models, Random Forest obtained the highest R^2^ in this evaluation (R^2^ = 0.991). However, the session-aware LOSO evaluation showed considerably greater variation in model performance. KNN achieved the lowest mean MAE and RMSE under LOSO validation, while the negative mean R^2^ values obtained by all evaluated models showed that the models did not generalize consistently between experimental sessions. These results show the importance of evaluating reconstruction models using independent measurement sessions rather than relying only on a random train–test split.

The potential energy benefit of UV reconstruction was also evaluated using a model-based analysis. With an estimated active power of 2.995 W and an acquisition duration of 10 s, one complete acquisition cycle requires approximately 0.00832 Wh. Under the assumption that each missing or invalid UV measurement would otherwise require one additional acquisition cycle, reconstructing the 75 unavailable UV values could avoid approximately 0.624 Wh of acquisition energy, corresponding to an estimated acquisition-related reduction of 4.94%. This value represents a potential saving under the assumptions used in the analysis and should not be interpreted as a directly measured reduction in total system power consumption or battery lifetime.

The current system was experimentally evaluated using one sensing node under urban monitoring conditions. Further work is therefore needed before the results can be extended to larger deployments or different water conditions. Future experiments will include additional sensing nodes, more independent acquisition sessions, longer field deployments, and evaluation under different water conditions. Lightweight models suitable for on-device reconstruction, as well as online model updating and anomaly detection, can also be investigated to improve the system in future deployments.

## Figures and Tables

**Figure 1:**
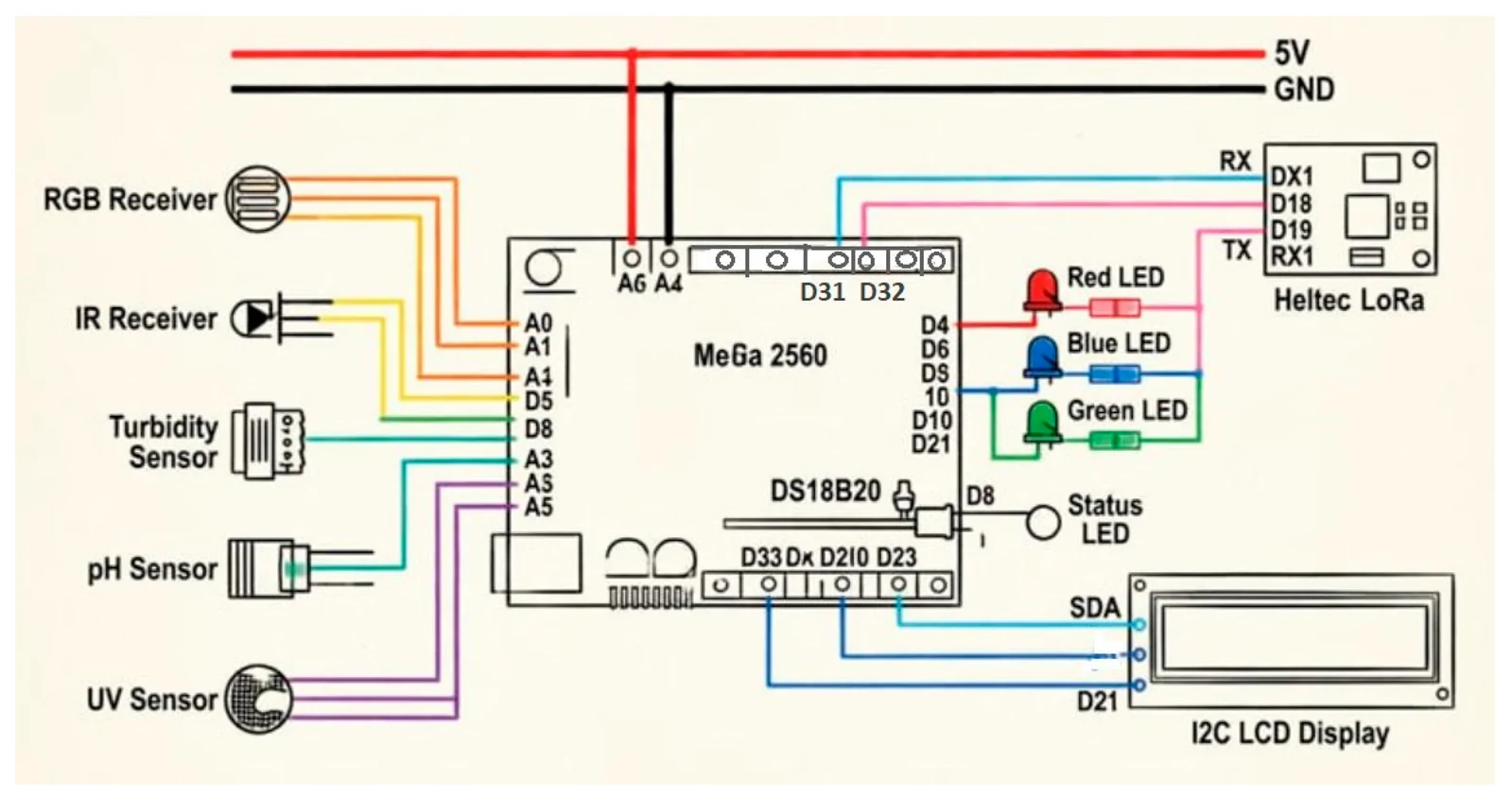
Schematic representation of the sensing-node enclosure and its main components.

**Figure 2:**
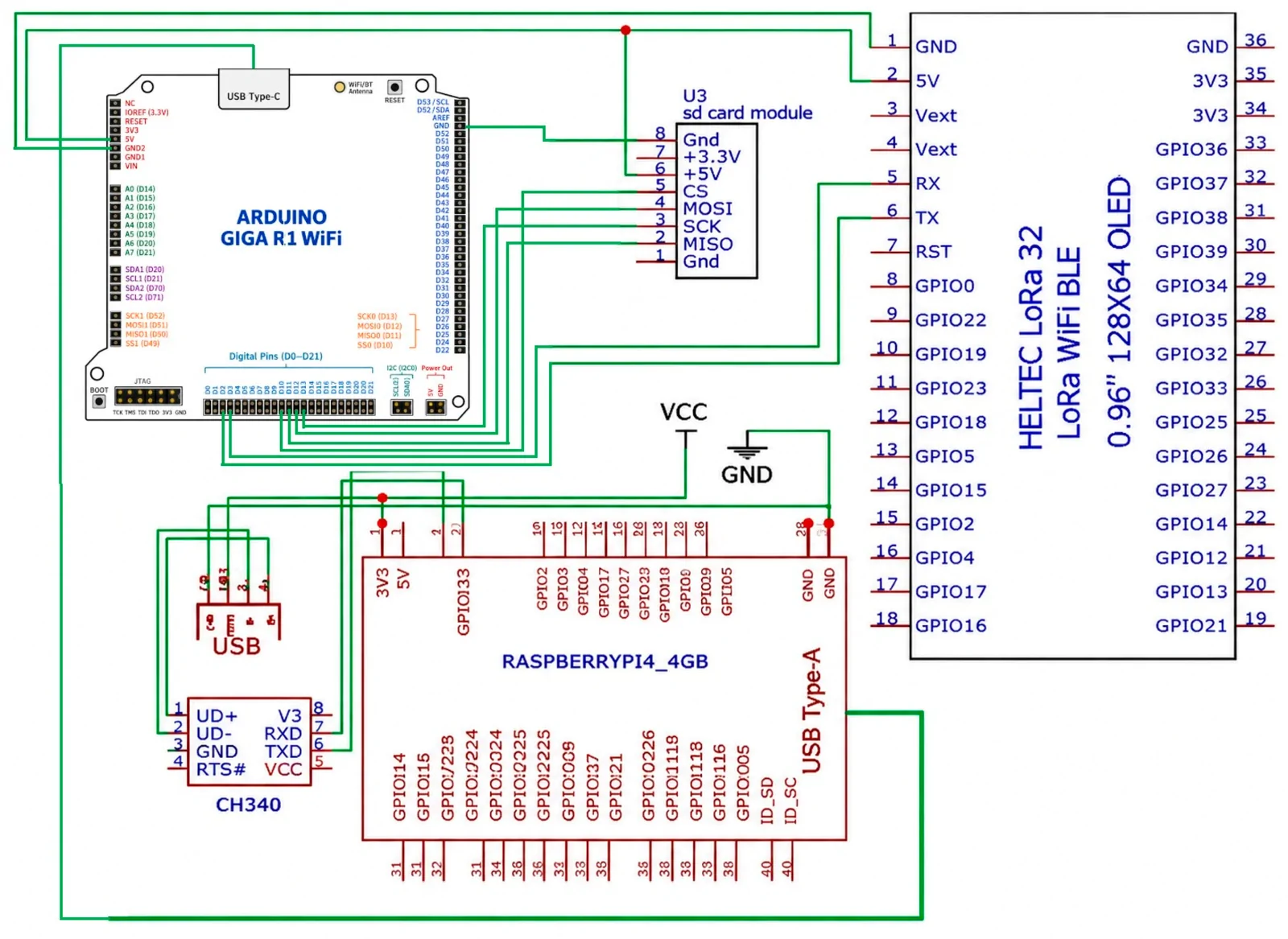
Schematic diagram of the gateway.

**Figure 3:**
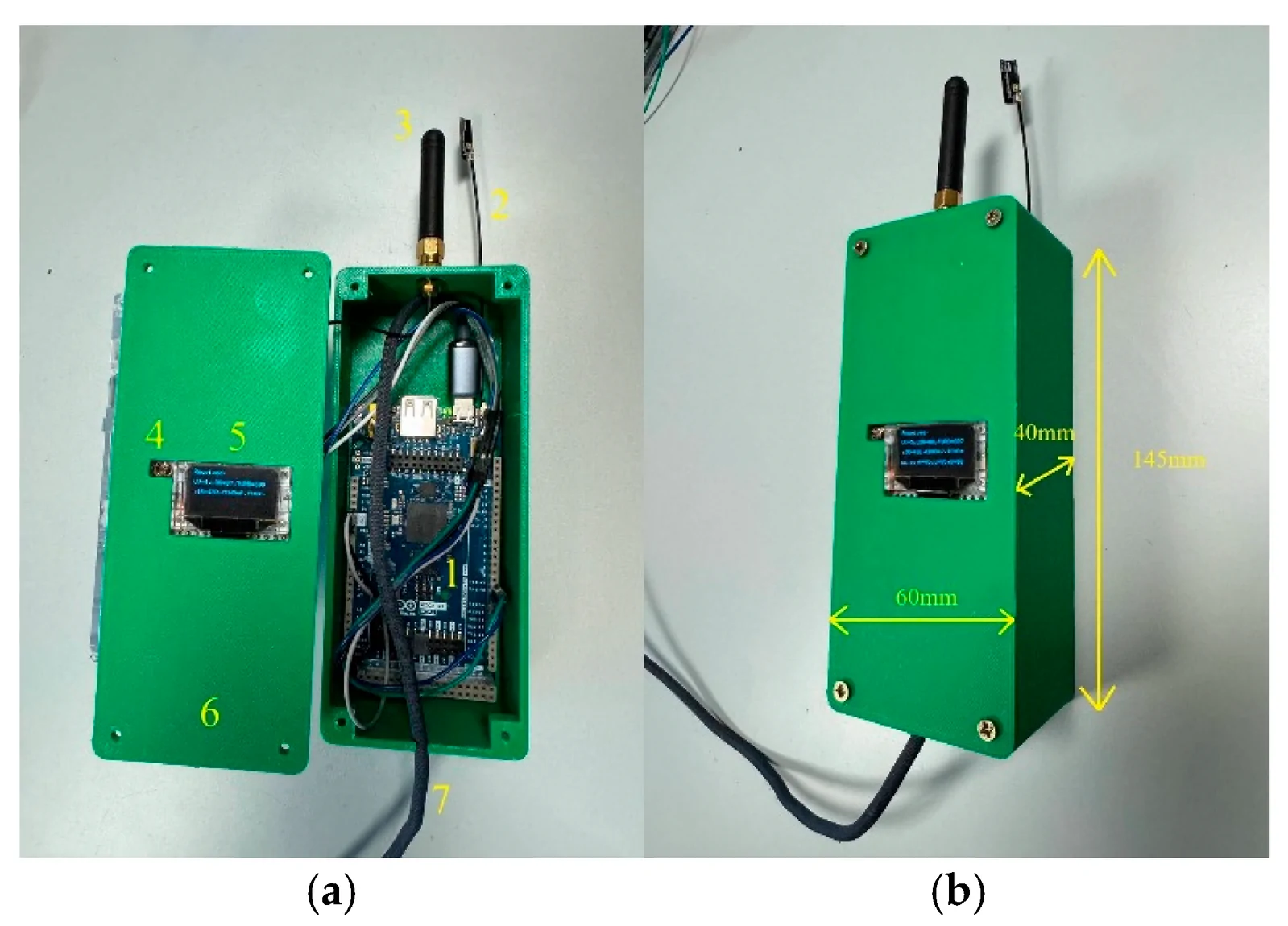
Proposed gateway: (**a**) External view of the implemented gateway showing the 3D-printed enclosure. (**b**) Internal view showing the Arduino GIGA, Heltec LoRa WiFi 868 V2 module, integrated OLED display, external antennas, and USB Type-C connection.

**Figure 4:**
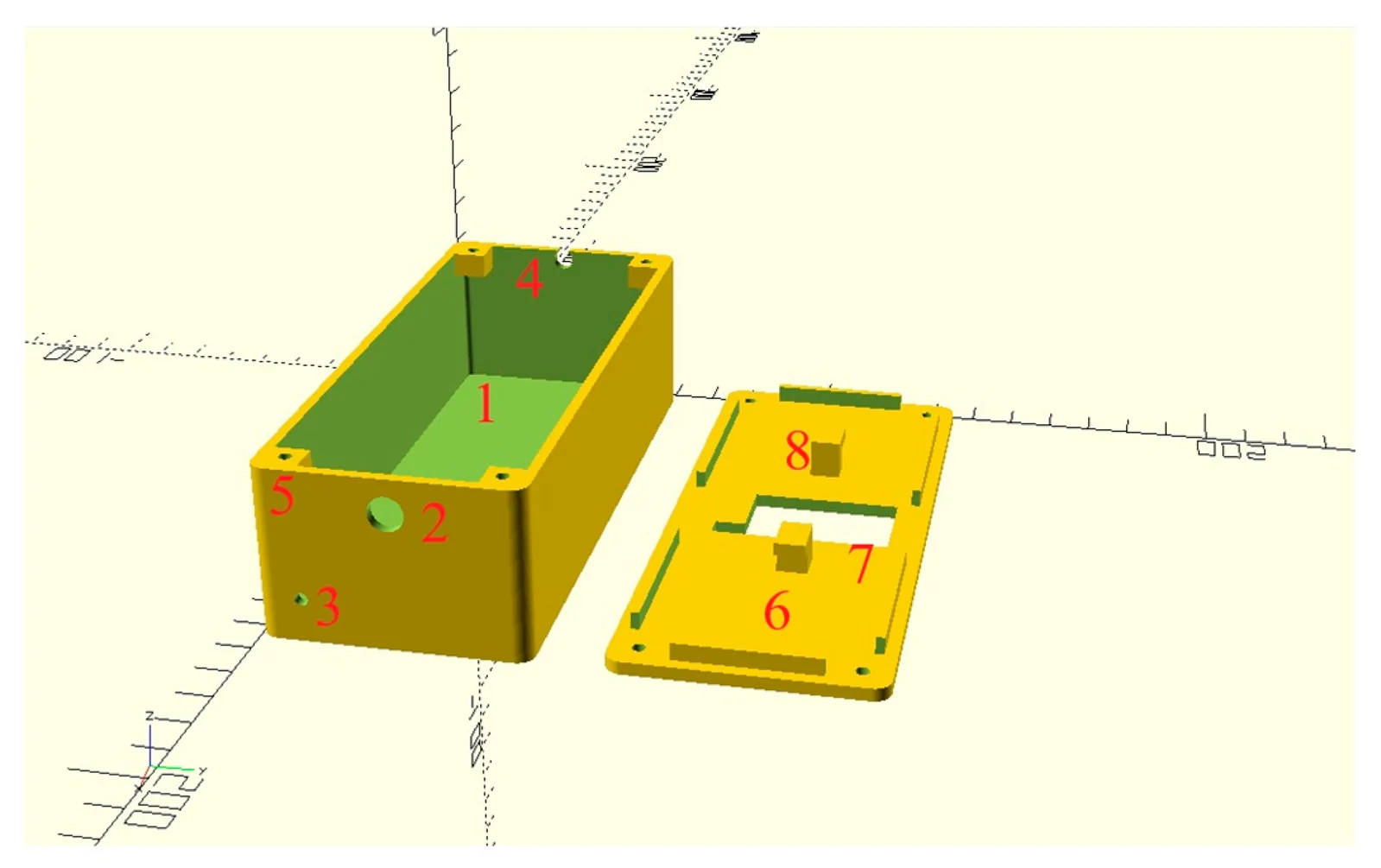
CAD model of the proposed 3D-printed gateway enclosure showing the main structural and mechanical features.

**Figure 5:**
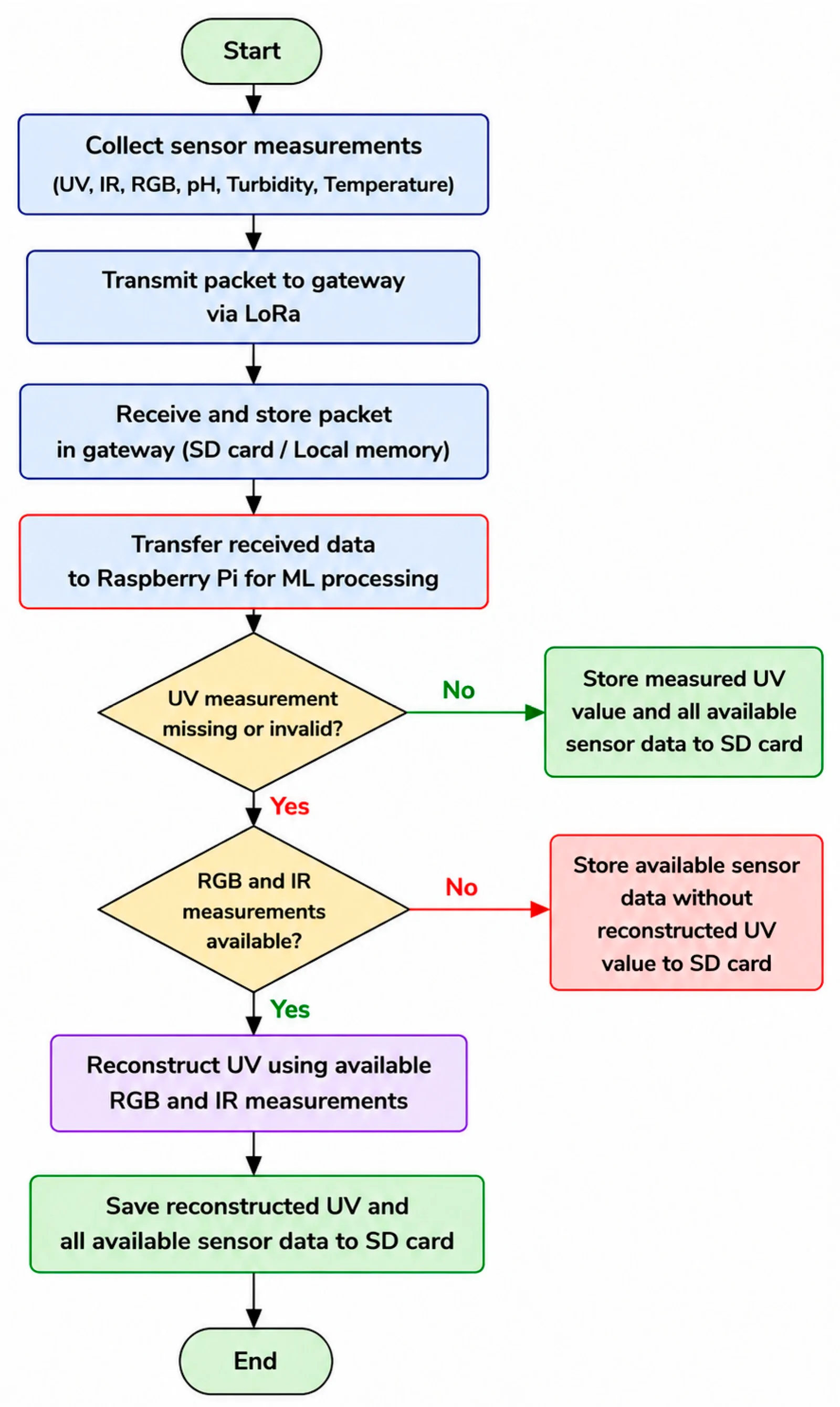
General system operation flowchart.

**Figure 6:**
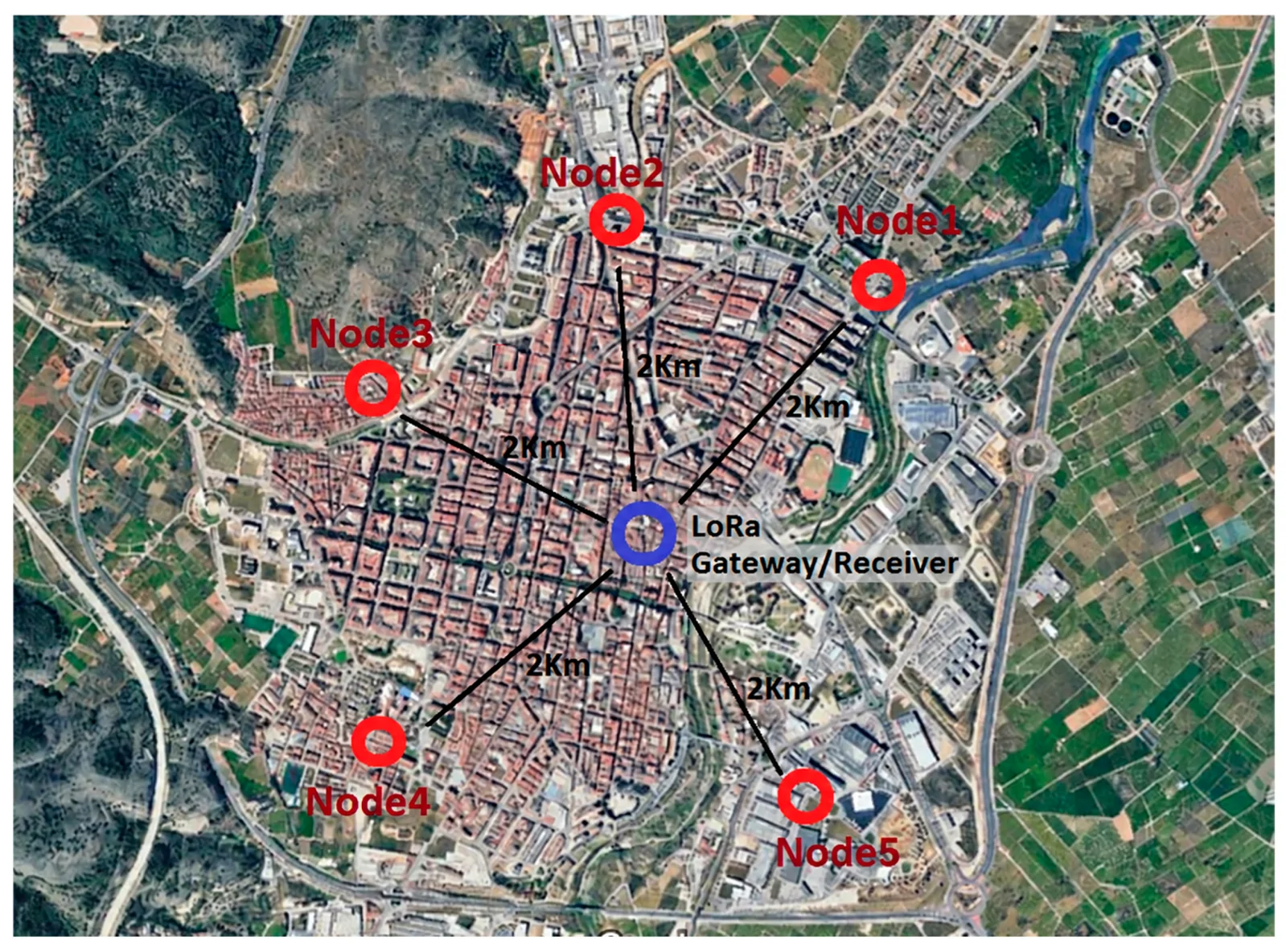
Point-to-point LoRa communication framework (868 MHz).

**Figure 7:**
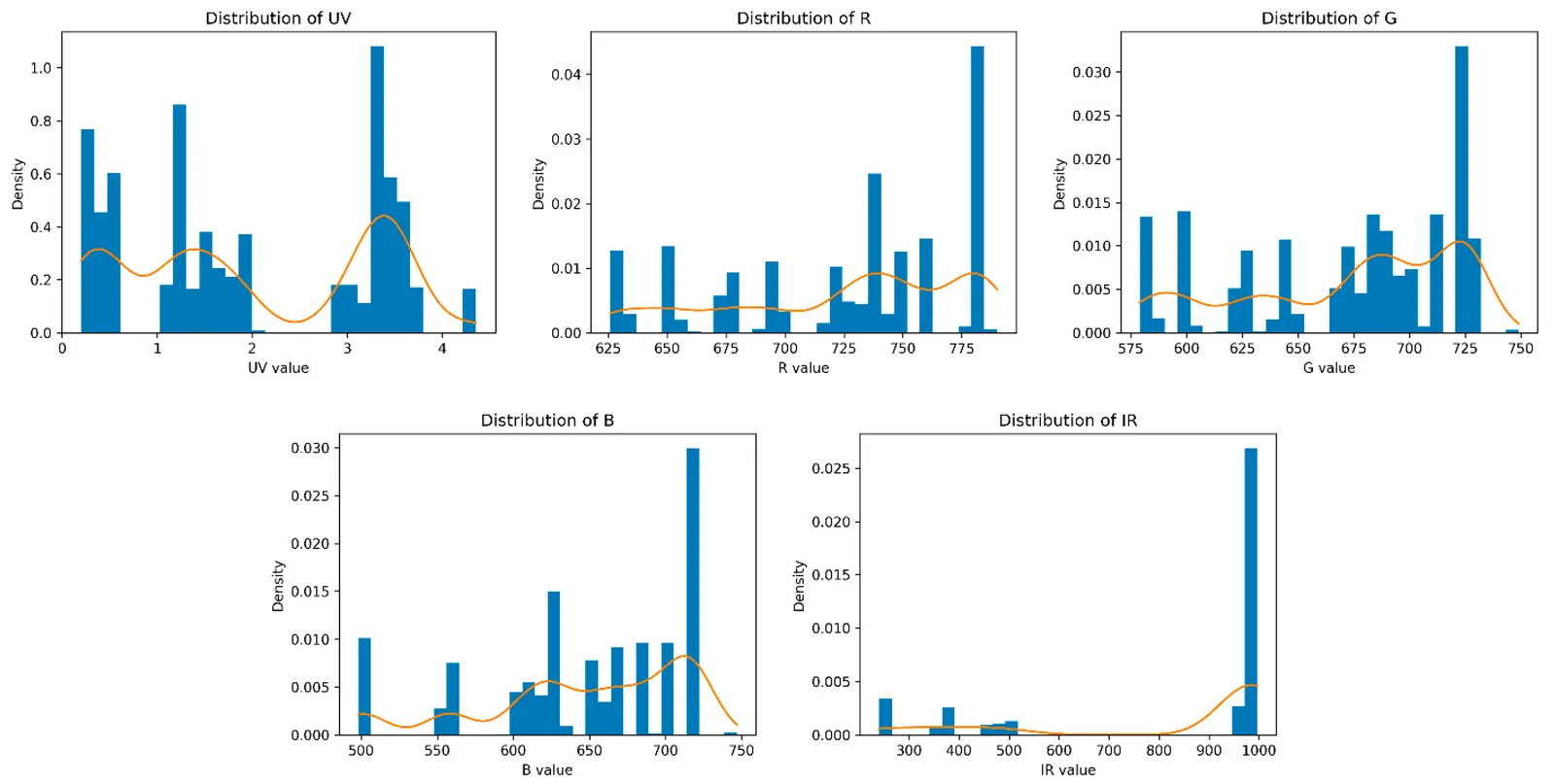
Distribution of sensor parameters for UV, R, G, B, and IR. The orange line represents the kernel density estimate (KDE) of each sensor parameter.

**Figure 8:**
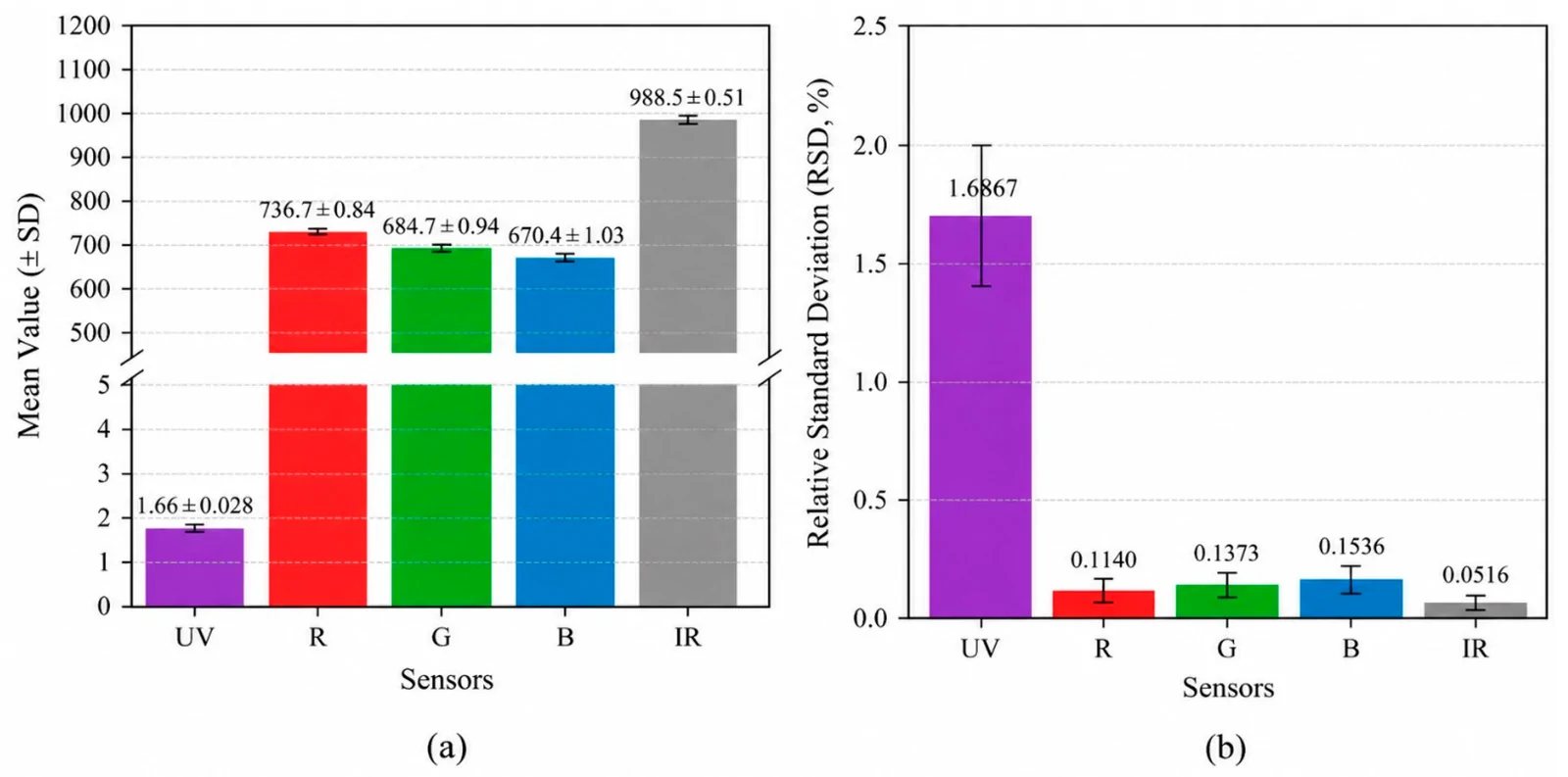
(**a**) Enhanced Visualization of Sensor Performance, (**b**) Standard Deviation. The different colors are used to distinguish the sensor parameters (UV, R, G, B, and IR).

**Figure 9:**
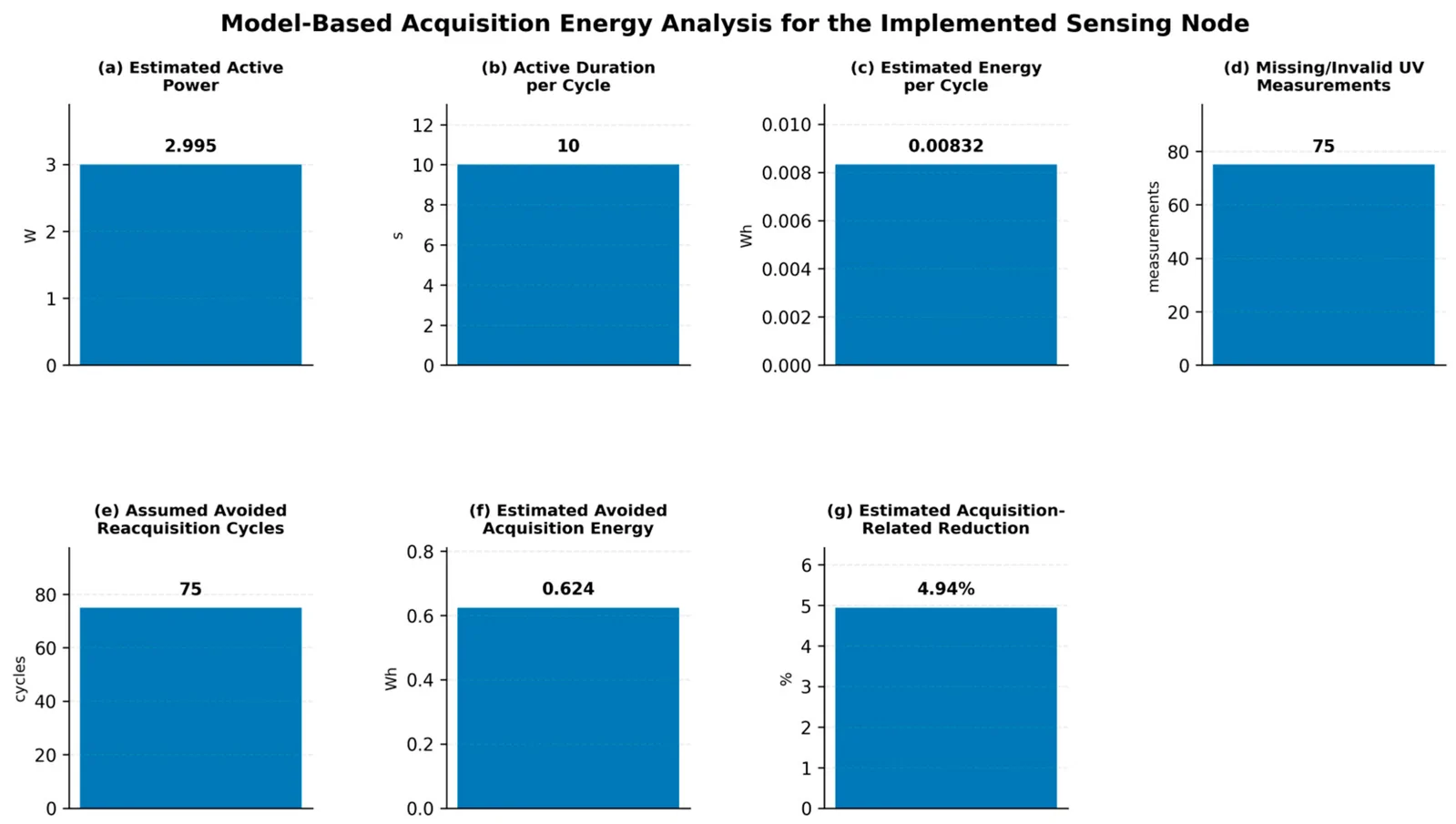
Model-based acquisition energy analysis for the implemented sensing node, including the estimated active power, active duration and energy per acquisition cycle, number of missing or invalid UV measurements, assumed avoided reacquisition cycles, estimated avoided acquisition energy, and estimated acquisition-related energy reduction.

**Figure 10:**
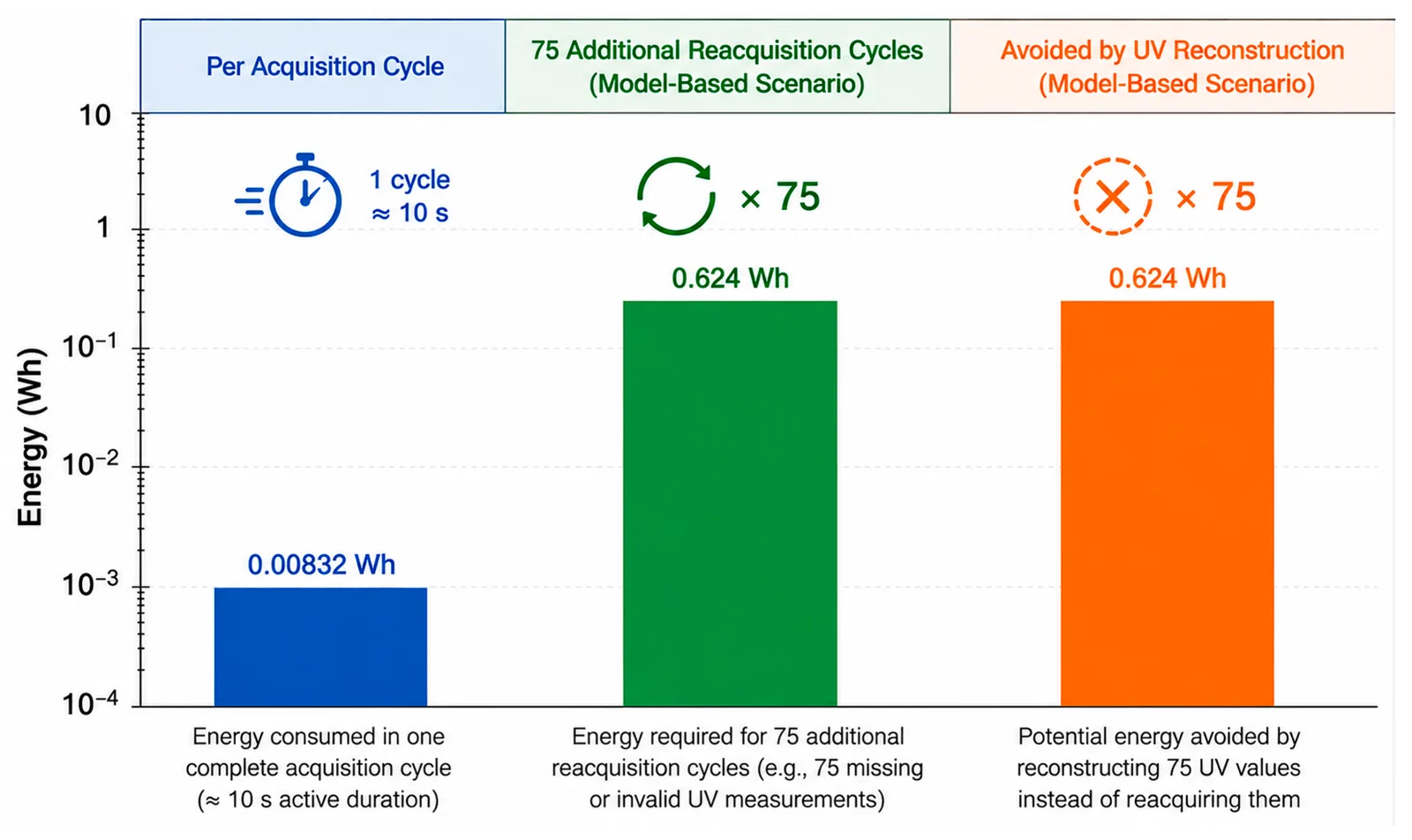
Model-based estimation of acquisition energy and potential energy saving obtained by avoiding additional acquisition cycles through UV reconstruction.

**Table 1:** Performance Comparison of Regression and Machine Learning Models.

Model	MSE (UV^2^)	R^2^
Multiple Linear Regression	0.001	0.12
Polynomial Regression (deg = 2)	0.00071	0.9633
SVR (RBF Kernel)	0.00044	0.978
KNN (k = 5)	0.00053	0.971
Random Forest	0.00022	0.991
MLP Neural Network	0.00036	0.983

**Table 2:** Performance of the evaluated models using Leave-One-Session-Out cross-validation.

Model	MAE (UV Units)	RMSE (UV Units)	R^2^
Multiple Linear Regression	2.832 ± 3.309	3.238 ± 3.983	−70.185 ± 95.730
Polynomial Regression (degree 2)	24.391 ± 46.400	33.362 ± 64.169	−10,628.454 ± 21,144.583
SVR (RBF Kernel)	0.486 ± 0.265	0.510 ± 0.259	−0.325 ± 1.050
KNN (k = 5)	0.306 ± 0.202	0.401 ± 0.317	−1.154 ± 3.567
Random Forest	0.347 ± 0.210	0.441 ± 0.301	−1.075 ± 3.133
MLP Neural Network	0.562 ± 0.365	0.597 ± 0.345	−37.003 ± 75.259

**Table 3:** Energy Consumption of Device Components.

Component	Power Consumption (W)
Arduino Mega	0.5
pH Sensor	0.1
IR Sensor	0.15
UV Sensor	0.15
RGB Sensor	0.25
Turbidity Sensor	0.2
Temperature Sensor	0.025
Heltec WiFi LoRa 868 V2	1.32
LCD (4 cm × 8 cm)	0.1
IR Receiver	0.05
UV Receiver	0.05
RGB Receiver	0.1
Raspberry Pi	12.5
SD Card Module	1
Arduino GIGA	0.8

**Table 4:** Estimated energy consumption and potential energy saving for Node 1.

Parameter	Estimated Value
Active power of Node 1	2.995 W
Active duration per acquisition cycle	10 s
Energy per acquisition cycle	0.00832 Wh
Total collected records	1443
Missing/reconstructed UV measurements	75 (5.2%)
Assumed avoided reacquisition cycles	75
Estimated avoided energy	0.624 Wh
Estimated acquisition-related energy reduction	4.94%
Nominal battery capacity	10,000 mAh (37 Wh at 3.7 V)
Avoided energy relative to nominal battery capacity	1.69%

**Table 5:** Estimated Active Power and Energy Consumption of the Gateway.

Parameter	Estimated Value
Active power consumption	15.32 W
Energy consumption for 1 h	15.32 Wh
Energy consumption for 5 h	76.60 Wh

**Table 6:** Estimated Active Power and Energy Consumption of the Complete System.

Parameter	Estimated Value
Total active power consumption	30.295 W
Energy consumption for 1 h	30.295 Wh
Energy consumption for 5 h	151.475 Wh

**Table 7:** Sensor Repeatability Statistics under Controlled Conditions.

Sensor	Mean	Standard Deviation	Min	Max	Relative Standard Deviation (RSD, %)
UV	1.66	0.028	1.61	1.71	1.699
R	736.7	0.844	734	738	0.115
G	684.65	0.938	681	686	0.137
B	670.43	1.026	666	672	0.153
IR	988.53	0.505	988	989	0.051
Temperature	16.42	0.014	16.41	16.43	0.086
pH	7.16	0.01	7.15	7.17	0.139
Turbidity	12	0	12	12	0

**Table 8:** Comparison of different research based on proposed models and their results.

Reference	Model Used	Data Used	Reported Performance	Practical Implementation
[13]	Not mentioned (Hardware focus)	Standard water quality parameters (e.g., pH, temperature, turbidity)	86%	Hardware implementation (LoRa), but no ML data reconstruction or missing data handling.
[18]	Not mentioned (Hardware focus)	Water environment parameters (e.g., pH, temperature, turbidity)	Not mentioned.	Hardware implementation (Distributed IoT/LoRa), but lacks edge-level ML predictive reconstruction.
[21]	Optical sensing (Hardware focus)	Optical properties (absorbance/transmittance) for drug detection	92%	Hardware implementation (Optical IoT sensor), but does not address missing data reconstruction via ML.
[24]	Not mentioned.	Turbidity, pH, Electrical Conductance, Nitrate, Arsenic, Fluoride, Dissolved O2	Not mentioned.	Only introducing the components required for implementation
[25]	Gradient boosting, polynomial regression, Multi-Layer Perceptron	Temperature, Turbidity, pH, Total Dissolved Solids	85%	Without any implementation
[26]	Artificial Neural Network, Support Vector Machine, regression, correlation analysis	Temperature, Turbidity, pH, Total Dissolved Solids	Not mentioned.	Without any implementation
[27]	Support Vector Machine, Random Forest, Logistic Regression, Decision Tree, CATboost, XGBoost, Multi-Layer Perceptron	pH, Electrical Conductance, Biochemical Oxygen Demand, Nitrate, Fecal Coliform, Total Coliform, Dissolved O2	94%	Without any implementation
[28]	J48, Naïve Bayes, Multi-Layer Perceptron	pH, Hardness, Total Dissolved Solids, Chloramines, Sulfate, Electrical Conductance, Trihalomethanes, Potability, Turbidity	Not mentioned.	Without any implementation
This paper	Linear Regression, Polynomial Regression, SVR, KNN, Random Forest, MLP Neural Network	Temperature, Turbidity, pH, IR, RGB, UV	R^2^ = 0.991 (random train–test evaluation)	Physical implementation and experimental evaluation of one sensing node

## Data Availability

The data, source code, electrical schematics, and CAD design files supporting the findings.

## Abbreviations

The following abbreviations are used in this manuscript:

AI	Artificial Intelligence
CatBoost	Categorical Boosting
DS18B20	Digital Temperature Sensor
EDA	Exploratory Data Analysis
IoT	Internet of Things
IR	Infrared
KNN	K-Nearest Neighbors
LCD	Liquid Crystal Display
LED	Light-Emitting Diode
LoRa	Long Range
ML	Machine Learning
MLP	Multi-Layer Perceptron
RF	Random Forest
RGB	Red, Green, and Blue
SD	Secure Digital
SVR	Support Vector Regression
UART	Universal Asynchronous Receiver–Transmitter
UV	Ultraviolet
WSN	Wireless Sensor Network
XGBoost	Extreme Gradient Boosting
